# Direct Dating and Physico-Chemical Analyses Cast Doubts on the Coexistence of Humans and Dwarf Hippos in Cyprus

**DOI:** 10.1371/journal.pone.0134429

**Published:** 2015-08-18

**Authors:** Antoine Zazzo, Matthieu Lebon, Anita Quiles, Ina Reiche, Jean-Denis Vigne

**Affiliations:** 1 Unité Mixte de Recherche 7209 ‘‘Archéozoologie, Archéobotanique: Sociétés, Pratiques et Environnements”, Centre National de la Recherche Scientifique, Muséum national d’Histoire naturelle, Sorbonne Universités, CP 56, 55 rue Buffon, F-75005, Paris, France; 2 Unité Mixte de Recherche 7194 « Histoire Naturelle de l’Homme Préhistorique » Centre National de la Recherche Scientifique, Muséum national d’Histoire naturelle, Sorbonne Universités, CP 56, 55 rue Buffon, F-75005, Paris, France; 3 Institut Français d’Archéologie Orientale, Pôle d’archéométrie, 37 rue al-Cheikh Aly Youssef B.P. Qasr el-Ayni 1152, 11441, Le Caire, Egypte; 4 Unité Mixte de Recherche 8220, Laboratoire d’Archéologie Moléculaire et Structurale, Centre National de la Recherche Scientifique, Université Pierre et Marie Curie, Sorbonne Universités, 4 place Jussieu, F-75005, Paris, France; University of Oxford, UNITED KINGDOM

## Abstract

In the Mediterranean, the island dwarf megafaunas became extinct around the end of the Pleistocene, during a period of rapid and global climate change. In Cyprus, this coincided with the first human presence on the island, as attested by the rock shelter of Akrotiri-*Aetokremnos* where an Epipaleolithic anthropogenic layer (stratum 2) was found overlying a massive accumulation of pygmy hippopotamus (*Phanourios minor* (Desmarest, 1822)) [Boekschoten and Sondaar, 1972] bones (stratum 4). The relationship between the two layers is highly controversial and the role played by humans in hippo extinction remains fiercely debated. Here, we provide new, direct radiocarbon and physico-chemical analyses on calcined bones which elucidates the complex depositional history of the assemblage. Bone turquoise was identified using micro-PIXE analysis and depth-profiling together with Vis spectroscopy, demonstrating that these bones were not freshly burned. Bayesian modeling of the radiocarbon dates indicates that stratum 4 accumulated during the first half of the 13^th^ mill cal BP and that calcination occurred several hundred years later. We conclude that accumulation occurred naturally during the beginning of the Younger Dryas and that Epipalaeolithic visitors subsequently used the bones as fuel, starting from the mid-13^th^ mill cal BP. At that time, dwarf hippos were probably already extinct or at least highly endangered. Our results shed new light on the possible causes of hippo extinction, on the subsequent introduction of the wild boar and on the earliest occupation of the island by humans.

## Introduction

Late Pleistocene and early Holocene megafauna extinctions are a global phenomenon the causes of which may have varied locally and are still widely debated [1,2,3]. Two scenarios are usually proposed. The global climate changes of the Younger Dryas or the rapid Holocene reheating have often been invoked as major factors. The other mechanism invoked for megafauna demise involves humans: either directly through hunting, indirectly through competition for resources, alteration of the natural environment or introduction of invasive species. These two mechanisms are not mutually exclusive. It is also possible that in some cases humans provoked the extinction of populations which were already weakened by climate change. Islands are perfect natural laboratories to study the impact of humans on the evolution of animal biodiversity [1,4,5]. In this context, Cyprus is central to the megafauna extinction debate.

Cyprus is a true oceanic island, since it has never been connected to any of the surrounding continents since the Messinian salinity crisis [6]. As a consequence of this isolation the composition of the Late Glacial Cypriot mammal communities is characterized by a reduced taxonomic diversity and a high degree of endemism. Extensive paleontological surveys indicate that the composition of the mammal fauna mainly included dwarf hippos (*Phanourios minor)*, dwarf elephants *(Elephas cypriotes)*, genets *(Genetta plesictoides)* and mice *(Mus cypriacus)* [7,8,9,10,11]. Among the surveyed localities, the site of Akrotiri-*Aetokremnos* stands out because it provided both the largest assemblage of the Cypriot dwarf megafauna and the earliest undisputable evidence of human presence on the island. Akrotiri-*Aetokremnos* is a small collapsed rock shelter located on the southern cliff of the Akrotiri peninsula on the island’s southern coast (S1 Fig). The site was excavated in the late 1980s and consisted of four distinct strata, two of which (stratum 2 and 4, respectively) have been interpreted by the lead excavator as major occupation layers [10]. Radiocarbon dating of charcoal from layers 2 and 4 attests that humans were present on Cyprus some 12,500 years ago. The upper layer (stratum 2) contains 88% of the cultural material recovered, including nine of the eleven identified archaeological features [10]. It provided an abundant lithic industry and food refuse, mainly composed of shellfish, fish and bird bones [10]. This layer also provided some dwarf hippo and elephant bones and the remains of small suids [12]. As there is no mention of suids in the Late Glacial Cypriot paleontological record, and taking into account the very low probability for natural colonisation, intentional introduction of the wild boar (*Sus scrofa*) by humans appeared to be the most parsimonious interpretation [12,13]. Stratum 2 is separated from the underlying stratum 4 by an intermittent sterile layer (stratum 3) consisting of sand blown from the dunes present between the cliff and the seashore, on the broad coastal plain created by the marine regression [14]. The lowest layer (stratum 4) is a dense bone accumulation containing 95% of the pygmy hippo remains and two archaeological features, both of which provided numerous burnt hippo bones.

Simmons [10] interpreted *Aetokremnos* as a processing site, and hypothesized that the accumulation of hippo and elephant bones was the result of human hunting. However, several arguments including the imbalance of bone and artefacts between these layers, the low level of fragmentation, the absence of cut marks [15] and possible mixing between stratum 2 and 4 [16,17] have led to other explanations arguing for a natural accumulation of bones followed by human occupation [14,16,18]. Nearly three decades after the initial publication of the site, there is still no agreement as to whether hippo extinction and human arrival are causally related [13,19,20,21]. The exact role played by humans in hippo extinction remains controversial, due to the problematic nature of the available bone dates. Bone collagen is not preserved, and most of the dates have been obtained from poorly characterized organic matter or the mineral fraction of unburnt bones. All but one are up to several thousands of years younger than the charcoal dates due to diagenetic alteration [22].

**Fig 1:**
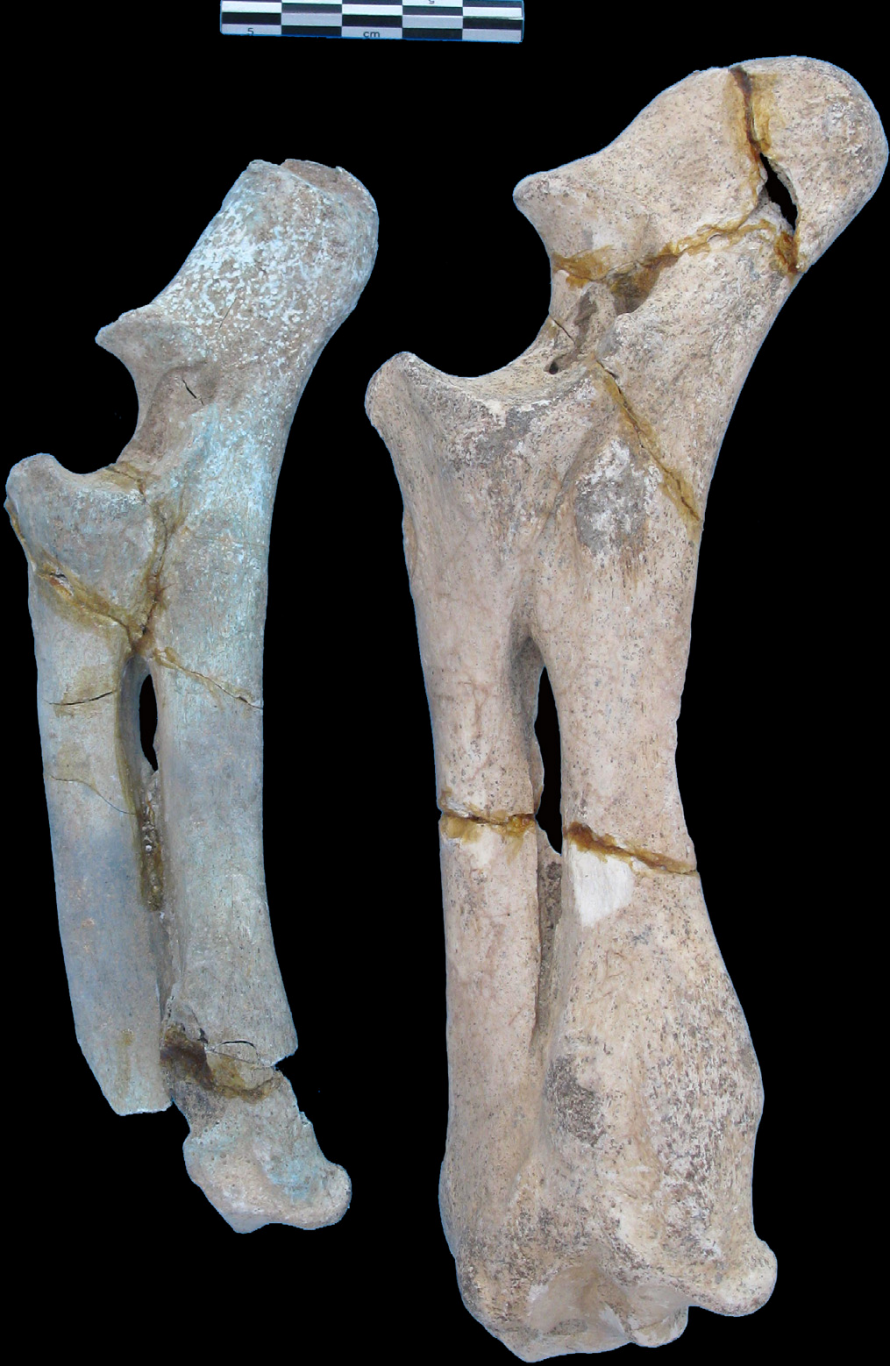
Lateral views of unburnt (right) and calcined (left) radio-ulna of adult dwarf hippos from Akrotiri-*Aetokremnos*. Both specimens belong to the species *Phanourios minor* (Desmarest, 1822) [Boekschoten and Sondaar, 1972] and come from stratum 4, Feature 3, FN684. The right specimen is unburned while the left specimen is calcined and shows the blue-green color. Note the smaller size of the calcined specimen (15% and 20–27% smaller for length and width, respectively) compared to the unburnt one. This difference in size mostly likely results from bone retraction due to calcination.

Our aim is to provide new insight into the extinction of the Cypriot endemic mega-fauna. In this paper, we report a series of 35 new radiocarbon determinations performed on hippo bones found in strata 2 and 4 (S2 Fig). Different fractions were dated, but the discussion focuses on calcined bone dates as this fraction has been shown to be extremely resistant to post-burial diagenetic contamination [23]. During sampling for dating, the presence of several intriguing pieces of pale blue-green colored bone, most of them belonging to feature 3 in stratum 4 (Fig 1) was observed. Their existence had been noted by the excavator but without further analysis [24]. Physico-chemical microanalyses and imaging by means of Fourier-transform Infrared spectroscopy (FTIR) and micro-Proton-induced X-ray Emission (micro-PIXE) were used to decipher the origin of the blue-green color of the Akrotiri bones and shed light into the taphonomic history of the bone assemblage.

## Results

### Physico-chemical analyses

A combination of different physico-chemical analyses, including FT-IR spectroscopy, carbon stable isotope analysis, micro-PIXE analysis and diffuse reflectance spectroscopy were performed to determine the timing and condition of heating of the calcined bones selected for dating. Heating causes an increase in the bone crystallinity [25] associated with a decrease in the carbon isotope value of the mineral fraction due to carbon isotope exchange with the fuel [26,27,28]. Carbon isotope values ranged between -20.7‰ and -26.8‰ which confirms that the white and blue-green bones are well calcined (Table 1). The Infrared Splitting Factor (IRSF) was measured using FT-IR spectroscopy to provide an estimation of the temperatures of calcination [29]. Values ranged between ca. 5 and 7, indicating moderate (600–650°C) to high (above 700°C) temperatures of calcination for all the bones (Table 1). We also looked for the presence of cyanamides in the calcined bones. Cyanamide formation (CN_2,_ absorption band at 2012 cm^-1^) is promoted by reducing conditions in the presence of ammonia, as can be the case when bones are heated in a hearth saturated with organic matter like wood, flesh or bones containing collagen [26,30,31]. No trace of cyanamide was found. Its absence in the calcined bones argues for the partial disappearance of organic matter from these bones before heating.

**Table 1:** IRSF values (FTIR), carbon isotope values and radiocarbon dates of the calcined bones and charcoal used in the Bayesian models.

Target #	Lab #	stratum	dated fraction	SF-FTIR	δ^13^C	^14^C age	error	calibrated range 2σ	
					(‰, VPDB)			from	to
P867/AA87182	AA8B2	surface	calcined bone apatite	-	-20.7	10485	57	12580	12126
P868/AA87183	AA14B1	2	calcined bone apatite	5.56	-24.4	10578	58	12684	12414
P869/AA87184	AA16B	2	calcined bone apatite	5.38	-25.2	10612	58	12705	12426
Muse154/SacA 28894	AA42B2	4a+b	calcined bone apatite	7.05	-23.8	10835	45	12791	12682
Muse152/SacA 28892	AA48B2	4a+b	calcined bone apatite	6.79	-26.8	10470	40	12559	12137
P935/AA88552	AA50B1	4a+b	calcined bone apatite	6.48	-23.6	10457	57	12557	12115
P870/AA87185	AA33B1	4b	calcined bone apatite	7.08	-22.8	10430	57	12539	12092
**average all hippos (n = 7)**						**10552**	**141**		
Beta-41002/ETH-7189		N96E89, st 2a lower	charcoal	-	-	10770	90	12829	12545
Beta-41408/ETH-7332		N96E89, st 2a	charcoal	-	-	10575	80	12715	12189
Beta-41406/ETH-7331		N97E88 st 2a	charcoal	-	-	10485	80	12638	12102
Beta-41000/ETH7188		N97E89, st 2a	charcoal	-	-	10420	85	12565	12015
OxA-15989		st 2	charcoal	-	-	10225	50	12136	11756
Beta-40382/ETH-7160		N97E89, st 4c	charcoal	-	-	10560	90	12707	12154
**average charcoal stratum 2 (n = 5)**						**10495**	**200**		

charcoal dates are from [10] and [36]; Dates are calibrated using Intcal 13 and Oxcal v4 software [45, 46]

**Fig 2:**
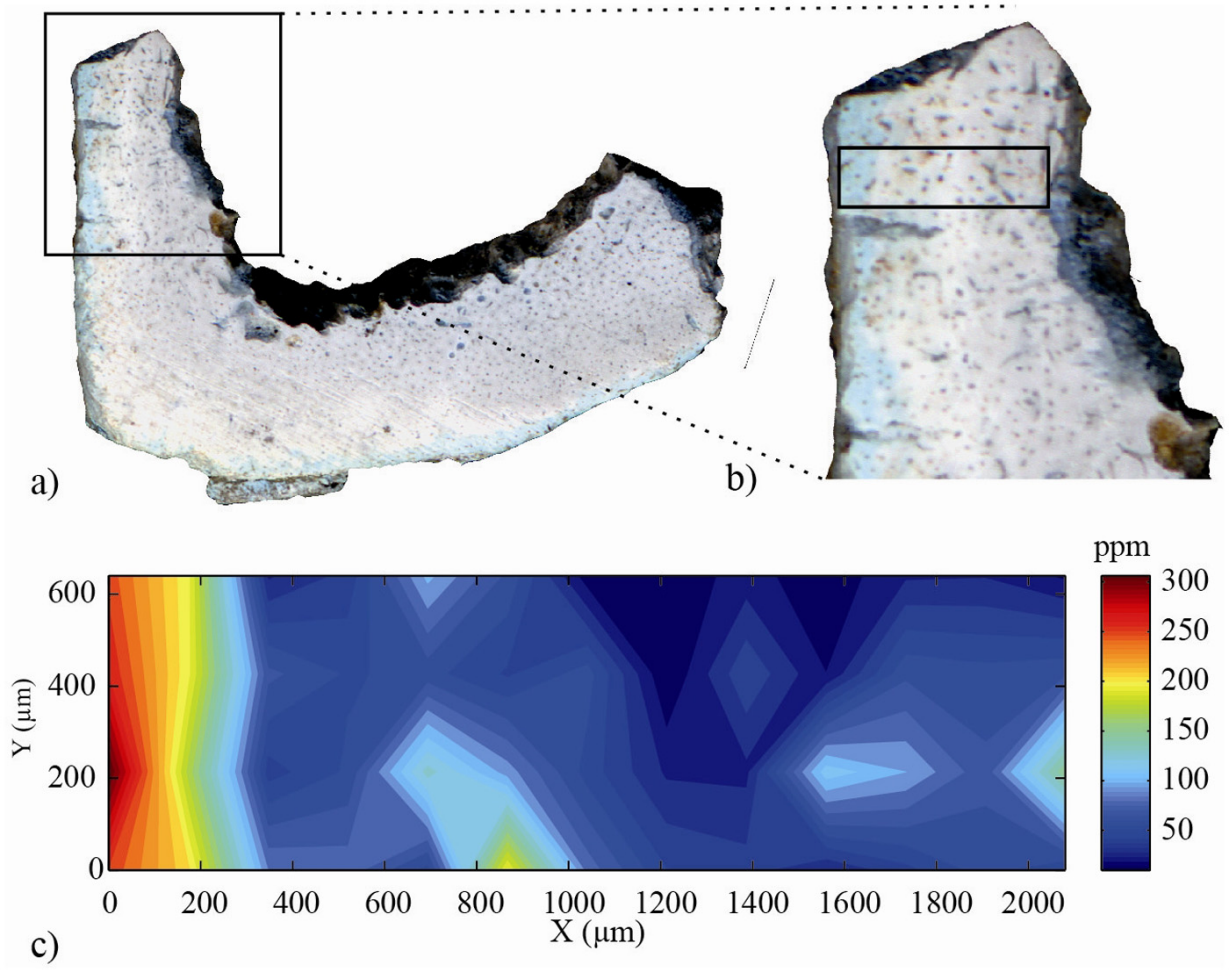
Optical view and distribution of manganese content over AA48 cross section. a) Optical view of the cross section of a long bone diaphyse (AA48); b) localization of elemental composition map; c) Distribution of manganese content over AA48 cross section. Manganese is mainly localized in the first 300 μm of the surface of the cortical bone.

Blue or green colored bones are often found on archaeological sites, but their origins can differ [32]. Micro-PIXE analysis indicates that copper (Cu) is under the detection limit in all the calcined samples (S1 Table), excluding Cu salts as a potential source of the blue color. In contrast, manganese (Mn) concentration varies from 124 to 3910 ppm for the studied samples (S1 Table). Mn concentration is higher in the blue-green areas located on the subsurface of the calcined bones than in the white (calcined) or charred (brown to black) areas. Diffuse reflectance spectroscopy demonstrates that Mn is incorporated in the crystalline structure of calcined bone apatite in the form of hypomanganate (MnO_4_^3-^) ions (S3 Fig). Hypomanganate ions are formed during exposure to temperatures above 500°C and are responsible for the blue-green color of the so called bone turquoise [32,33,34]. Modern hippo teeth are devoid of Mn and therefore the presence of hypomanganate ions in the apatite structure attests to a Mn uptake prior to heating. The presence of bone turquoise in stratum 4 demonstrates that the assemblage must have undergone post depositional uptake of manganese and was diagenetically altered when heated [32]. Cross sections of two different calcined bones were also analyzed by micro-PIXE to characterize spatially the elemental uptake within these samples (Fig 2 and S4 Fig). Manganese incorporation is well correlated with the blue coloration and is restricted to the subsurface of the samples (first 300–500μm).

### Radiocarbon dating

A total of thirty five radiocarbon measurements were produced on fifteen different bones and teeth (S1 Text, S2 Table, S4 and S5 Figs). The dated fractions included the decomposed collagen from burnt bones and the biogenic carbonate present in bone, tooth enamel and dentine apatite, as well as in calcined bone apatite. The results indicate a large dispersion of the radiocarbon ages (from 10,835±45 BP to 6,347±45 BP). With the notable exception of the calcined bones, all of these fractions yielded radiocarbon ages that were younger and, considered altogether, more variable than the average charcoal date (10,506±181 BP, n = 6). This finding confirms previous results obtained on a smaller set of burnt and unburnt hippo bones [22], and suggests that the soluble and insoluble organic fractions from burnt bones are contaminated by organic soil carbon, while dentine and enamel apatite are contaminated by soil dissolved bicarbonate. Conversely, calcined bone dates are tightly clustered and range between 10,430±57 and 10,835±45 BP. Their average (10,552±141 BP, n = 7) is not significantly different from the charcoal age (t test: t[uneq. var.] = 0.51, p[Mt Carlo] = 0.38). This result confirms that calcined bone is highly resistant to post-depositional carbon isotopic exchange [23,35].

Bayesian modeling was applied to further constrain the timing of stratum 2 and 4 deposition. The dataset (Table 1) is composed of the seven calcined bone samples and the previously published AMS dates on charcoal [22,36]. Two models were tested (S7–S9 Figs). Model 1 (S7 Fig) follows [10] and considers that the hippo bones from stratum 2 were actually deposited together with this stratum, implying that they survived until the date of human occupation of the shelter, dated to ca. 12,500 cal BP. Model 2 (S8 Fig) follows [14,16,18] and considers that all the bones found in upper stratum 2 result from post-depositional mixing and actually came from stratum 4, implying that extinction took place before the deposition of stratum 2. In this model, all the hippo bone dates were grouped in the same phase (stratum 4). Both models produced very similar results (Table 2) but their overall agreement (Am) was low, 11% and 23%, respectively. In each of the two models, two charcoal dates (all in stratum 2) with a low individual agreement index were removed from the subsequent analysis and the models were run again. They produced an acceptable agreement (Am) of 75% and 85%, respectively. The final age models and boundaries generated are shown in Table 2. Both models agree to place the formation of stratum 4 during the first half of the 13^th^ mill cal BP and the beginning of stratum 2 during the second half of the 13^th^ mill cal BP. The main difference between them lies in the duration of stratum 2. According to Model 1, the formation of stratum 2 is rather short and ends before the end of the 13^th^ mill cal BP. The boundaries of the second model are less sharp, and suggest that human occupation lasted until the first half of the 12^th^ mill cal BP. As both models are equally supported, Bayesian statistics alone do not allow us to conclude whether the hippo bones found in stratum 2 are in a primary context.

**Table 2:** Probability distribution (at two sigmas) for the start and end boundaries of stratum 2 and 4 and for the duration of the three strata in Models 1 and 2.

		Stratum 4			Stratum 3	Stratum 2		
Model	Amodel	start	end	Duration	duration	start	end	duration
		yr, cal BP	yr, cal BP	Yrs	yrs	yr, cal BP	yr, cal BP	yrs
Model 1	11	12893–12660	12523–12446	166–414	0–50	12524–12446	12471–12007	0–466
	75	12891–12656	12522–12439	163–424	0–56	12500–12425	12483–12358	0–100
Model 2	23	12865–12654	12525–12308	161–500	0–175	12508–12230	12405–11812	0–619
	86	12887–12672	12501–12219	210–600	0–286	12432–12081	12371–11642	0–678

## Discussion

For the first time, a coherent series of radiocarbon dates allowed the direct dating of hippo bones in Akrotiri-*Aetokremnos* to the first half of the 13^th^ millennium cal BP. The identification of bone turquoise on several specimens from the lowermost layer (stratum 4), together with the absence of cyanamide in all the calcined bones analyzed indicates that the assemblage has a complex depositional history, and that bone accumulation and heating are clearly separated in time.

Several lines of evidence suggest that the amount of time separating bone accumulation and burning was rather short on a geological timescale. First, the abundance of charred (black) bones in the assemblage indicates that the bones still contained organic matter when they were burned. Second, Mn incorporation is rather superficial as indicated by micro-PIXE profiles. It is likely that the Mn uptake corresponds to the outer portion of the bone whose organic matter had already disappeared when the bone was burned. The available literature suggests that it is possible to relate Mn incorporation with time, at least semi-quantitatively. Very low Mn concentrations (25 ppm) have been measured at the surface of bone samples from the Second World War burials [37]. On the contrary, high (ca. 1000 ppm) and consistent levels have been measured for well preserved bones from the Neolithic [38]. Intermediate although variable (50–300 ppm) concentrations were measured for more recent bones dating from the medieval period [39]. Although many aspects such as the Mn content of the local bedrock, hydrology and climate can act on bone diagenesis and Mn diffusion rate, these data suggest that the Akrotiri bones could have spent several centuries in the ground prior to burning. The final evidence comes from the radiometric estimates themselves: the difference in radiocarbon activity between calcined bones and charcoal being less than 2% modern carbon (pMC). It is worth remembering here that carbon isotope exchange occurs between bone and the combustion atmosphere during calcination [26,28,40]. As a result, only a small fraction of the carbon present in a calcined bone originates from the bone itself, most of it comes from the fuel. Open air experiments showed that calcination of archaeological samples with modern wood systematically resulted in an apparently younger age of the bone apatite carbonate due to the replacement of 70–90% of bone inorganic carbon by wood carbon [28]. If we consider a replacement of 70–90% of bone carbon by wood carbon, a simple mass balance calculation allows estimating the initial difference in ^14^C activity at less than 7 pMC, which translates into a difference of about 580 radiocarbon years. These estimates are in accordance with the prediction of the two Bayesian models tested, which suggest that stratum 4 accumulated for 150–600 years prior to burning (Table 2). This result confirms the conclusions of a previous taphonomic study where evidence for post-depositional fracture was found [15]. While this finding does not completely exclude the role of humans in the accumulation of the bone assemblage at an earlier time, this is clearly not the most parsimonious scenario given the absence of cut marks already noted by the same author [15]. Rather, our results give more strength to the scenario suggesting that hippo bones in stratum 4 accumulated naturally as has happened at numerous localities around the coast of Cyprus [41]. This accumulation could have resulted from the trapping of animals in an open-roofed cave. Aven-trapping of several tens or hundreds of individuals is a common process of terrestrial vertebrate accumulation in karstic areas [42,43], which have been documented elsewhere in Cyprus [8,11]. But most of the rock shelters that formed in the sea cliffs of the Akrotiri peninsula are associated with the Nicosia formation, which is not karstic, and the stratigraphy of Akrotiri-*Aetokremnos* matches that of a collapsed rockshelter [44]. Therefore, accumulation likely results from the frequentation of the cave over several hundreds of years by pygmy hippos searching for fresh water and protection from the heat [41]. It could represent a palimpsest of catastrophic die-offs of the endemic fauna due to shortage of water and food under drought conditions at the beginning of the Younger Dryas [16].

The data presented here are pertinent to the debate regarding the exact timing of human arrival on the island. Stratum 4 has been interpreted as an occupation layer [10] to some criticism [13,14,16,18]. It is noteworthy that some of the dated bone turquoise was found in feature 3, a burnt fauna concentration spread over 6 m^2^ where chipped stone was also found. Feature 3 (S2 Fig) was sealed by sterile layer (stratum 3) in its western end where the calcined bones originated. The sterile layer did not show any trace of heating, and thus, accidental burning from a hearth situated above, or bone migration for this part of the site can be excluded. Direct contact with the fire is necessary to calcine a bone [45]. This suggests that human populations could have been already present on the island before the deposition of the sterile sandy stratum 3. Epipalaeolithic people who visited the island during the middle of the Younger Dryas probably used the bone bed as a source of fuel at a time when wood was scarce. The cavity was then briefly abandoned as evidenced by the sporadic deposition of the sterile layer overlying stratum 4. Bayesian modeling of the radiocarbon dates on charcoal from stratum 2 indicates that site occupation continued during the second half of the 13^th^ millennium cal BP and may have lasted until the first half of the 12^th^ millennium cal BP. During that time, humans processed food, as evidenced by the numerous shells and bird remains present in stratum 2 and may have continued to extract fossil hippo bones for fuel from the underlying stratum 4, as evidenced by the presence of several pits cross-cutting the stratigraphy [10].

Whether hippos were still present at the time of stratum 2 deposition cannot be inferred based on the radiometric evidence, as neither of the two Bayesian models proved conclusive. It is probable, however, that the endemic fauna was already extinct or at least highly endangered when humans arrived on the island, as evidenced by the imbalance of hippo bones between strata 2 and 4. Cross sections published for the site show that stratum 3 is scarce in the rear part of the shelter where disturbed layers are mentioned [10]. In addition, stratum 3 is cut by several pits elsewhere in the site with a refit being noted between an *Elephas* specimen found in Stratum 1/2 and one near the bottom of Stratum 4 [17]. It is therefore possible that the hippo bones found in stratum 2 may actually come from lower in the stratigraphy [16]. Pertinent to this question is the presence of eighteen suid remains most of them found in stratum 2. This indicates that by the mid-13^th^ mill cal BP, wild boar had already been introduced from the Near-East onto the island [12]. The presence of this first invasive species makes more sense in a context where wild game is lacking locally. Wild boars would have been free to occupy the niche left vacant by the hippos, while providing abundant and easy to catch game for the Epipalaeolithic and early Neolithic seafarers [13].

## Material and Methods

### Sample selection

Fifty six samples (AA1 to AA56, S1 Text) coming from the site of Akrotiri-*Aetokremno*s (S1 Fig) were sampled in May 2009 by two of the authors (A.Z and J.-D.V) for radiocarbon dating and physico-chemical analyses. Samples were on loan from the Kourion Museum in Episkopi (Cyprus) where they are curated and permanently held. An export permit (n° 14.01.015.011) was delivered by the Department of Antiquities of Cyprus for these samples (S1 and S2 Files). The archaeological bones reported in this paper are temporarily deposited for study in the Archaeozoology, Archaeobotany laboratory from the Muséum national d’Histoire naturelle, Paris, and will be finally deposited in the Kourion Museum at Episkopi, Cyprus. Among them, fifteen were selected for radiocarbon dating. Most of them were burnt to various degrees from charred to calcined. They represent the different layers of the site: surface (n = 1), strata 2 (n = 2), strata 2/4 (n = 1), and strata 4 a+b (n = 5), strata b (n = 5) and strata c (n = 1). Their position in the stratigraphy is indicated in S2 Fig.

### Radiocarbon dating

Collagen, when preserved, is the most reliable support for bone radiocarbon dating. Radiocarbon dating of hippo bones in *Aetokremnos* proved challenging because climatic conditions do not favor bone collagen preservation, a situation also observed in early Holocene Cypriot sites like *Klimonas* or *Shillourokambos*. Because the direct dating of hippo bones is critical we explored the possibility of dating the other carbon bearing fractions (both organic and mineral) present in the hippo bones. When bones are burned at a low temperature (ca. 300°C) collagen decomposes and reduced carbon becomes trapped in its mineral (apatite) structure. This organic carbon can be extracted following an acid-alkali-acid pre-treatment, similar to the protocol used for charcoal. This is done by using HCl (1N), followed by NaOH (0.125 N) then HCl (1N) again. In order to monitor possible contamination we dated both the alkali (NaOH) insoluble and the alkali soluble fraction where possible. Inorganic carbon from biogenic carbonate present in bone, dentine and enamel was also dated. Enamel is the material of choice for stable isotope analysis as its high crystallinity and low porosity which makes it resistant to diagenetic alteration. Isolated attempts at dating the carbonate fraction of tooth enamel have been previously published with limited success. Finally, carbonate present in calcined bones (i.e. heated above 600°C) was also dated. Carbonate remaining in calcined bones is believed to be extremely resistant to diagenetic alteraction, and has been shown to produce reliable dates, at least for the Holocene. Alkali-soluble and insoluble organic carbon was combusted at 800°C in the presence of O_2_ and the evolved CO_2_ was trapped by cryogeny. Apatite samples were powdered then pre-treated under vacuum in acetic acid to remove secondary carbonates. Pre-treated powders were then reacted in 4 ml of orthophosphoric acid at 90°C and CO_2_ was trapped by cryogeny. Graphitisation, AMS measurements and data processing were performed at the University of Arizona AMS ^14^C lab (Tucson, AZ) and at the Artemis Facility of Gif-sur-Yvette (France). Two different protocols were tested on four calcined bone and two tooth enamel samples to investigate whether duration and strength of the acetic acid pre-treatment had an influence on the quality of the dates. Duplicates were either treated in 1N acetic acid for a short period of time (5–8 h) or in 2N acetic acid for 24h. We saw no major difference in the measured ages, suggesting that the light treatment was successful in removing all the secondary calcite present in the sample. The dates were calibrated using Intcal 13 and the Oxcal software [46,47]. Only the oldest age of the pair was used for the Bayesian model because it was considered the best approximation of the pristine ^14^C age for that sample (S1 Text).

### FTIR analysis

Fourier transform infrared spectrometry (FTIR) analysis was performed in transmission mode using KBr pellet technique. KBr pellets were prepared by mixing KBr with bone powder (grain size < 2.5%) at a concentration of .25%. Infrared spectra were acquired on a Vector 22 FTIR spectrometer (Bruker) by co-adding 64 scans with a spectral resolution of 2 cm^-1^. The relative carbonate content was estimated using the absorbance ratio of ν3 CO_3_ at 1415 cm^-1^ and ν3 PO_4_ band at 1045 cm^-1^ (CO_3_/PO_4_). The crystallinity of bone mineral was measured using the infrared splitting factor (IRSF) following published protocols [25]. The presence of cyanamides (CN_2_) in bone apatite was monitored from their absorption band at 2012 cm^-1^.

### Micro-PIXE analysis

Elemental micro-analysis and profiles were carried out using the 3 MeV external proton micro-beam of “AGLAE”, the tandem particle accelerator facility of C2RMF in the Louvre Museum (Paris—France). Details of AGLAE facility are described in Calligaro et al. [48]. Elemental concentration, limit of detection and statistical were computed using GUPIX software and combining high and low energy spectrum acquired by the two Si(Li)-detectors [49]. Major, minor and trace composition elemental composition were achieved locally focusing external proton beam (50 μm in diameter) at the surface bone samples. Areas between 100 to 500 μm were analyzed. Particular attention was paid to turquoise/blue areas that were suspected to contain manganese ions (hypomanganate; MnO_4_^3-^). This compound is characteristic of bones that have been buried for sufficient time in sediment to allow the uptake of manganese before being heated at temperature over 600°C (S2 Text). Calcined bone cross sections were scanned with different moving step from 100 to 500 μm to achieve profiles of elemental composition (S2 Table).

### Visible diffuse reflectance

Diffuse reflectance spectrophotometry was applied to samples showing blue coloration and compared to reference spectra of hypomanganate containing apatite (S3 Fig). The portable spectrophotogoniometer of the Centre for Research and Restoration of the French Museums (C2RMF, Paris) was used for the investigations. This STIL spectrometer uses light from a 100W electric bulb together with fibre optics to illuminate the probed area at a set angle (such as 45°). The diffuse reflected light was collected at the same angle by the same fibre and passed into the spectrometer. Spectra were collected in the range of 400 to 800 nm.

## Supporting Information

S1 File Export request Letter by Alan Simmons (Univ Nevada, USA). (PDF)

S2 File Export permit by the Department of Antiquities (Lefkosia, Cyprus). (PDF)

S3 File Permission to publish Fig 1. (PDF)

S1 Text Radiocarbon dating. (DOC)

S2 Text Identification of bone turquoise. (DOC)

S3 Text Cited references. (DOC)

S1 Table Sample list. (DOC)

S2 Table Manganese and cupper contents on bone surface measured by μPIXE analyses. (DOC)

S3 Table List of the thirty five radiocarbon measurements on hippo bones from Akrotiri-*Aetokremnos*. (DOC)

S1 Fig Location of the site of Akrotiri-*Aetokremnos*, Cyprus. (TIF)

S2 Fig Location of the dated samples. (DOC)

S3 Fig Vis spectra showing the presence of hypomanganate ions in the apatite structure. (DOC)

S4 Fig Optical view and distribution of manganese content over AA50 cross section. (DOC)

S5 Fig Calibrated dates for the thirty five bone samples from Akrotiri-*Aetokremnos*. (DOC)

S6 Fig Intra-individual variability in radiocarbon age measured for different mineral and organic fractions of a partly burnt hippopotamus mandible (AA30). (DOC)

S7 Fig First Bayesian model (Model 1) for the radiocarbon dates obtained at the Akrotiri-*Aetokremnos* sequence. (DOC)

S8 Fig Second Bayesian model (Model 2) for the radiocarbon dates obtained at the Akrotiri-*Aetokremnos* sequence. (DOC)

S9 Fig Projection along the calibration curve of the calibrated radiocarbon likelihoods determinations and posterior probabilities used for Model 2. (DOC)
