# Supplementary material for: Direct Dating and Physico-Chemical Analyses Cast Doubts on the Coexistence of Humans and Dwarf Hippos in Cyprus
Source: PLoS One. 2015 Aug 18;10(8):e0134429. doi: 10.1371/journal.pone.0134429 (PMC4540316; doi:10.1371/journal.pone.0134429)
Supplement: S3 File — (PDF) [file pone.0134429.s003.pdf]

Institut National ECOLOGIE ET ENVIRONNEMENT du CNRS
Département « ÉCOLOGIE ET GESTION DE LA BIODIVERSITE » du Muséum
(UMR 7209 / USM 303) Bâtiment 56, 55, rue Buffon, F-75005 Paris, France

Paris, 7/8/2015

I give permission for the open-access journal PLOS ONE to publish the figure #1 of the paper submitted by Zazzo et al., untitled : "Lateral views of adult two radio-ulna of *Phanourios minor* (Desmarest, 1822) from Akrotiri-*Aetokremnos* (stratum 4, Feature 3, FN684)" under the Creative Commons Attribution License (CCAL) CC BY 4.0 (http://creativecommons.org/licenses/by/4.0/). I am aware that this license allows unrestricted use and distribution, even commercially, by third parties."

Jean-Denis Vigne
DR CNRS

☎. : 33 (0)1 40 79 33 10 / 38 51 **fax**. 33 (0)1 40 79 33 14 **email** : vigne@mnhn.fr
