# Supplementary material for: Direct Dating and Physico-Chemical Analyses Cast Doubts on the Coexistence of Humans and Dwarf Hippos in Cyprus
Source: PLoS One. 2015 Aug 18;10(8):e0134429. doi: 10.1371/journal.pone.0134429 (PMC4540316; doi:10.1371/journal.pone.0134429)
Supplement: S1 Text — (DOC) [file pone.0134429.s004.doc]

**S1 Text. Radiocarbon dating**

A total of thirty five radiocarbon measurements were produced from fifteen different bones and teeth (Table S2). The dated fractions included the decomposed collagen from burnt bones and the biogenic carbonate present in bone, tooth enamel and dentine apatite, as well as in calcined bone apatite. The AMS dates from charcoal published for the site (1, 2) were used as a benchmark, samples younger than the average charcoal age (10,506±181 BP, n= 6) were considered unreliable. The results indicate a large dispersion of radiocarbon ages, from 10,835±45 BP to 6,347±45 BP (Table S2). When different fractions of the same specimen are dated, the age dispersion ranges can vary from 100 to 4,000 BP (Fig. S6). The youngest dates were measured using the insoluble organic carbon from charred bones (9,189±1240 BP, n= 10), followed by dentine apatite (9,667±559 BP, n= 3), the soluble organic fraction of charred bones (10,006±368 BP, n= 4) and enamel apatite (10,156±396 BP, n= 5). With the notable exception of one enamel date (10,565±45 BP, SacA28887) all of these fractions yielded radiocarbon ages that were both younger and more variable than the average charcoal date. This finding confirms previous results obtained on a smaller set of burnt and unburnt hippo bones (3), and suggests that the soluble and insoluble organic fractions from burnt bones are contaminated by organic soil carbon, while dentine and enamel apatite are contaminated by soil dissolved bicarbonate. Doubts have been expressed by other authors on the reliability of dates obtained on charred bones (4). The results from this large scale survey, with regards to bone and enamel apatite dates, showed that the exchange of carbon between bioapatites and the burial environment takes place quickly (5), and always leads to increased levels of ^14^C in the carbonate phase. The effect of diagenesis on the ^14^C age of the apatite samples is documented in all types of bioapatite, not just in bone, and can be quite large in late Pleistocene samples.

Previous studies have opposed this, however, demonstrating that calcined bone is actually highly resistant to the post-depositional carbon isotopic exchange (6, 7). Dates obtained from the seven calcined hippo bone fragments are tightly clustered and range between 10,835±45 BP and 6,347±45 BP. Their average (10,552±141 BP, n= 7) is not significantly different from the charcoal age (p= 0.61). Only calcined bones dates were used in the Bayesian models. Duplicates were run on three different samples to test the accuracy of the pretreatment procedure. No systematic difference was observed. However, some of the duplicates did not pass the Chi2 test. Only the oldest age of each pair was used in the Bayesian model. This was motivated by two arguments: (1) a slight age shift towards a younger age cannot totally be excluded for Late Palaeolithic bones (8); (2) carbon isotope exchange between wood and bone occurs during calcination (9, 10). In the case where a paleontological deposit is used as fuel, this will also shift the bone age toward a younger age (11). Therefore, the oldest age is probably closer to the original age of the bone sample.
