# Supplementary material for: Direct Dating and Physico-Chemical Analyses Cast Doubts on the Coexistence of Humans and Dwarf Hippos in Cyprus
Source: PLoS One. 2015 Aug 18;10(8):e0134429. doi: 10.1371/journal.pone.0134429 (PMC4540316; doi:10.1371/journal.pone.0134429)
Supplement: S2 Text — (DOC) [file pone.0134429.s005.doc]

**S2 Text. Identification of bone turquoise**

Turquoise colored bones, also called *odontolite* or *bone turquoise* are one of the most fascinating imitations of mineral turquoise used in the Middles Ages as a semi-precious stone to decorate art and religious objects. Former spectroscopic studies demonstrated that its chemical composition corresponds to apatite containing trace amounts of Fe, Mn, Ba and sometimes U with Fe- and Mn-oxides on the bone surface. Modern bones are generally devoid of these elements and oxides that are introduced or deposited diagenetically. Bone turquoise can be formed after a heat treatment of diagenetically altered bones bearing Mn traces to temperatures in the range of 500 to 800 °C (12, 13).

Diffuse reflectance spectroscopy allowed identifying characteristic absorption patterns for bone turquoise (315, 410, 600, 640, 680, 760, 900, 1170 nm) corresponding to the same spectroscopic features as blue geological apatites and ivory bearing Mn^5+^ traces. Mn^5+^ ions (3d^2^) in an oxygenated tetrahedral environment are the coloring species in these apatites. X-ray absorption spectroscopy (XANES and EXAFS) as well as time-resolved laser-induced luminescence spectroscopy (TRLIF) determined that Mn is essentially in the oxidation state 2+ in fossils or altered bone before the heat process, in addition to the presence of localized Mn oxides (Mn^3+/4+^). After heat treatment, Mn is principally in the oxidation state 5+. These Mn^5+^ traces substitute the phosphate ions, which when combined in an oxygenated tetrahedral environment allows the production of the intense blue color of bone turquoise, through a ligand-field splitting of the external electronic levels (12-14).

The large absorption band in the region of 600 to 700 nm corresponds to the electronic transition (^3^A_2_🡪 ^3^T_1_ (^3^F)) of Mn5+ ions due to the absorption of red light. This absorption pattern can therefore be used to identify the color origin of bone turquoise, as is the case for the bone turquoise found in the lower stratum of Akrotiri-*Aetokremnos*.
