# Supplementary material for: Direct Dating and Physico-Chemical Analyses Cast Doubts on the Coexistence of Humans and Dwarf Hippos in Cyprus
Source: PLoS One. 2015 Aug 18;10(8):e0134429. doi: 10.1371/journal.pone.0134429 (PMC4540316; doi:10.1371/journal.pone.0134429)
Supplement: S3 Text — (DOC) [file pone.0134429.s006.doc]

**S3_Text. Cited References**

1. Simmons A & Mandel R (2007) Not such a new light: A response to Ammerman and Noller. *World Archaeology* 39(4):475-482.

2. Simmons AH & et al. (1999) *Faunal extinction in an island society: pygmy hippopotamus hunters of Cyprus* pp i-xxi, 1-381.

3. Wigand P & Simmons AH (1999) The dating of Akrotiri Aetokremnos. *Faunal extinction in an island society*, ed Simmons AH ((Kluwer Academic / PLenum Press, New York, Boston), pp 193-216.

4. Higham T*, et al.* (2011) Precision dating of the Palaeolithic: A new radiocarbon chronology for the Abri Pataud (France), a key Aurignacian sequence. *J. Hum. Evol.* 61(5):549-563.

5. Zazzo A (2014) Bone and enamel carbonate diagenesis: A radiocarbon prospective. *Palaeogeogr., Palaeoclimatol., Palaeoecol.* 416(0):168-178.

6. Lanting JN, Aerts-Bijma AT, & van der Plicht J (2001) Dating of cremated bones. *Radiocarbon* 43(2A):249-254.

7. Zazzo A & Saliege JF (2011) Radiocarbon dating of biological apatites: A review. *Palaeogeography Palaeoclimatology Palaeoecology* 310(1-2):52-61.

8. Zazzo A*, et al.* (2013) Can We Use Calcined Bones for C-14 Dating the Paleolithic? *Radiocarbon* 55(2-3):1409-1421.

9. Huls CM, Erlenkeuser H, Nadeau MJ, Grootes PM, & Andersen N (2010) Experimental Study on the Origin of Cremated Bone Apatite Carbon. *Radiocarbon* 52(2):587-599.

10. Van Strydonck M, Boudin M, & De Mulder G (2010) The Carbon Origin of Structural Carbonate in Bone Apatite of Cremated Bones. *Radiocarbon* 52(2):578-586.

11. Zazzo A, Saliege JF, Lebon M, Lepetz S, & Moreau C (2012) Radiocarbon Dating of Calcined Bones: Insights from Combustion Experiments under Natural Conditions. *Radiocarbon* 54(3-4):855-866.

12. Reiche I*, et al.* (2001) From mastodon ivory to gemstone: The origin of turquoise color in odontolite. *Am. Mineral.* 86(11-12):1519-1524.

13. Reiche I, Vignaud C, & Menu M (2000) Heat induced transformation of fossil mastodon ivory into turquoise 'odontolite'. Structural and elemental characterisation. *Solid State Sciences* 2(6):625-636.

14. Borromei R, Oleari L, & Day P (1981) Electronic spectrum of the manganate(V) ion in different host lattices. *Journal of the Chemical Society, Faraday Transactions 2: Molecular and Chemical Physics* 77(9):1563-1578.

15. Bronk Ramsey C (2009) Bayesian analysis of radiocarbon dates. *Radiocarbon* 51(1):337-360.

16. Reimer PJ*, et al.* (2013) IntCal13 and Marine13 Radiocarbon Age Calibration Curves 0-50,000 Years cal BP. *Radiocarbon* 55(4):1869-1887.
