# Supplementary material for: Direct Dating and Physico-Chemical Analyses Cast Doubts on the Coexistence of Humans and Dwarf Hippos in Cyprus
Source: PLoS One. 2015 Aug 18;10(8):e0134429. doi: 10.1371/journal.pone.0134429 (PMC4540316; doi:10.1371/journal.pone.0134429)
Supplement: S1 Table — (DOC) [file pone.0134429.s007.doc]

**Table S1. Sample List**

The list of exported samples is given below. They comprise fifty-six bone and tooth fragments coming from twenty-four different contexts. The archaeological bones reported in this paper are temporarily deposited for study in the Archaeozoology, Archaeobotany laboratory of the Muséum national d’Histoire naturelle, Paris, and will be finally deposited in the Kourion Museum at Episkopi, Cyprus.

| **bag #** | **sample ID** | **Level** | **Provenience** | **anatomical determination** | **thermal state** | **mass (g)** |
| --- | --- | --- | --- | --- | --- | --- |
| 1 | AA1 | FN 72 | surface, west scree section | bone fragment | burnt, partly calcined (<10% white) | 5 |
| 1 | AA2 | FN 72 | surface, west scree section | bone fragment | calcined, white to blue | 8 |
| 1 | AA3 | FN 72 | surface, west scree section | bone fragment | inner burnt, outer calcined (10-15%) | 3 |
| 1 | AA4 | FN 72 | surface, west scree section | bone fragment | 100% calcined, white to blue | 6 |
| 1 | AA5 | FN 72 | surface, west scree section | bone fragment | 80% calcined | 7 |
| 1 | AA6 | FN 72 | surface, west scree section | bone fragment | inner charred, outer calcined (50/50) | 5 |
| 2 | AA7 | FN 72 | surface, west scree section | bone fragment | inner charred, outer calcined (50/50) | 19 |
| 2 | AA8 | FN 72 | surface, west scree section | bone fragment | inner charred, outer calcined (50/50) | 27 |
| 2 | AA9 | FN 72 | surface, west scree section | bone fragment | calcined, white (not sure it is calcined) | 6 |
| 2 | AA10 | FN 72 | surface, west scree section | bone fragment | calcined, white | 3 |
| 3 | AA11 | FN 121 | N95E88, lv. 4b | canine fragment | non burnt | 9 |
| 3 | AA12 | FN 121 | N95E88, lv. 4b | canine | charred? Chocolate-black colour inside | 18 |
| 4 | AA13 | FN 125 | N98E88/87, lv. 2/4,-80cm to bedrock | distal humerus fragment | partly charred (30%) | 54 |
| 5 | AA14 | FN 208 | N94E91, lv. 2a | proximal tibia | 90% calcined | 115 |
| 6 | AA15 | FN 208 | N94E91, lv. 2a | calcaneum | calcined, white | 24 |
| 7 | AA16 | FN 208 | N94E91, lv. 2a | bone fragment | calcined, white | 9 |
| 7 | AA17 | FN 208 | N94E91, lv. 2a | bone fragment | calcined, white | 6 |
| 8 | AA18 | FN 270 | N93E8, lv. 4b | bone fragment | calcined, white | 8 |
| 8 | AA19 | FN 270 | N93E8, lv. 4b | bone fragment | calcined, white | 7 |
| 8 | AA20 | FN 270 | N93E8, lv. 4b | bone fragment | calcined, white | 2 |
| 8 | AA21 | FN 270 | N93E8, lv. 4b | bone fragment | partly burnt | 3 |
| 8 | AA22 | FN 270 | N93E8, lv. 4b | bone fragment | partly burnt | 1 |
| 8 | AA23 | FN 270 | N93E8, lv. 4b | bone fragment | partly burnt | 4 |
| 8 | AA24 | FN 270 | N93E8, lv. 4b | bone fragment | partly burnt | 3 |
| 9 | AA25 | FN 271 | N94E89, lv. 4b | mandible fragment with two teeth | unburnt | >200 |
| 9 | AA26 | FN 271 | N94E89, lv. 4b | mandible fragment with two teeth | unburnt | 45 |
| 9 | AA27 | FN 271 | N94E89, lv. 4b | mandible fragment with two teeth | unburnt | 67 |
| 10 | AA28 | FN 306 | N94E90, lv. 4b | distal radius | calcined | 33 |
| 11 | AA29 | FN 374 | N93E90, lv. 4b | mandible with fragmented teeth | partly charred (dentine, mandibular bone) | 105 |
| 11 | AA30 | FN 374 | N93E90, lv. 4b | mandible with fragmented teeth | partly burnt | 78 |
| 11 | AA31 | FN 374 | N93E90, lv. 4b | mandible with fragmented teeth | partly burnt | 45 |
| 12 | AA32 | FN 375 | N94E90, lv. 4b | maxillar fragment | unburnt | 94 |
| 13 | AA33 | FN 375 | N94E90, lv. 4b | bone fragment | outer calcined, inner burnt | 40 |
| 14 | AA34 | FN 451 | N99E87, lv. 4b | maxillar fragment | unburnt | 74 |
| 14 | AA35 | FN 451 | N99E87, lv. 4b | maxillar fragment encolle | unburnt | 64 |
| 14 | AA36 | FN 451 | N99E87, lv. 4b | maxillar fragment | unburnt | 35 |
| 15 | AA37 | FN 683 | N95E89, lv. 4b | petrosal | 50% charred | 8 |
| 15 | AA38 | FN 683 | N95E89, lv. 4b | petrosal | calcined? | 8 |
| 15 | AA39 | FN 683 | N95E89, lv. 4b | petrosal | calcined | 9 |
| 16 | AA40 | FN 684 | N97E88, lv. 4b eastern 1/2 | bone fragment | partly burnt | 17 |
| 16 | AA41 | FN 684 | N97E88, lv. 4b eastern 1/2 | bone fragment | partly burnt | 7 |
| 16 | AA42 | FN 684 | N97E88, lv. 4b eastern 1/2 | bone fragment | calcined, partly green | 10 |
| 16 | AA43 | FN 684 | N97E88, lv. 4b eastern 1/2 | bone fragment | calcined, inner charred (<5%?) | 9 |
| 17 | AA44 | FN 684 | N97E88, lv. 4b eastern 1/2 | tooth | burnt | 9 |
| 17 | AA45 | FN 684 | N97E88, lv. 4b eastern 1/2 | tooth | burnt | 11 |
| 17 | AA46 | FN 684 | N97E88, lv. 4b eastern 1/2 | tooth | burnt | 6 |
| 18 | AA47 | FN 684 | N97E88, lv. 4b eastern 1/2 | fragment of tibia | burnt (internal surface only) | 44 |
| 19 | AA48 | FN 684 | N97E88, lv. 4b eastern 1/2 | fragment of tibia | calcined, partly green | 50 |
| 20 | AA49 | FN 684 | N97E88, lv. 4b eastern 1/2 | fragment of humerus | calcined | 31 |
| 21 | AA50 | FN 684 | N97E88, lv. 4b eastern 1/2 | fragment of humerus | charred (internal surface), calcined (outer half) | 40 |
| 22 | AA51 | FN 684 | N97E88, lv. 4b eastern 1/2 | fragment of femur | charred (inner half), calcined (outer half) | 19 |
| 23 | AA52 | FN 767 | N95E88, lv. 4c | fragment of bone | partly burnt | 3 |
| 23 | AA53 | FN 767 | N95E88, lv. 4c | fragment of bone | partly burnt | 4 |
| 23 | AA54 | FN 767 | N95E88, lv. 4c | fragment of bone | partly burnt | 2 |
| 23 | AA55 | FN 767 | N95E88, lv. 4c | fragment of bone | partly burnt | 1 |
| 24 | AA56 | FN 767 | N95E88, lv. 4c | fragment of ulna | charred (inner half), calcined (outer half) | 50 |
