# Supplementary material for: Direct Dating and Physico-Chemical Analyses Cast Doubts on the Coexistence of Humans and Dwarf Hippos in Cyprus
Source: PLoS One. 2015 Aug 18;10(8):e0134429. doi: 10.1371/journal.pone.0134429 (PMC4540316; doi:10.1371/journal.pone.0134429)
Supplement: S2 Table — (DOC) [file pone.0134429.s008.doc]

**Table S1.** Manganese and cupper contents (in ppm) on bone surface measured by µPIXE analyses (“- “: below the limit of detection)

| **Samples** | **MnO K** | **CuO K** |
| --- | --- | --- |
| AA33 - grey area | 634 | - |
| AA33 - turquoise area | 2850 | - |
| AA33 - turquoise area | 3054 | - |
| AA39 - white area | 309 | - |
| AA39 - white area | 124 | - |
| AA39 - bluish area | 242 | - |
| AA42 - brown area | 1499 | 44 |
| AA42 - light blue area | 1391 | - |
| AA42 - turquoise area | 2150 | - |
| AA42 - turquoise area | 1692 | - |
| AA43 – turquoise area | 1000 | - |
| AA43 - turquoise area | 1175 | - |
| AA43 - internal carbonized part | *-* | - |
| AA43 - internal carbonized part | - | *-* |
| AA43 - porous area | 843 | - |
| AA48 - turquoise area | 3897 | - |
| AA48 - turquoise area | 2562 | - |
| AA48 - turquoise area | 3910 | - |
| AA50 - turquoise area | 1576 | *-* |
| AA50 - turquoise area | 1789 | - |
| AA51 - porous turquoise area | 2697 | - |
| AA51 - porous turquoise area | 3120 | - |
| Modern hippo tusk Cyprus | - | - |
