# Supplementary material for: Direct Dating and Physico-Chemical Analyses Cast Doubts on the Coexistence of Humans and Dwarf Hippos in Cyprus
Source: PLoS One. 2015 Aug 18;10(8):e0134429. doi: 10.1371/journal.pone.0134429 (PMC4540316; doi:10.1371/journal.pone.0134429)
Supplement: S3 Table — (DOC) [file pone.0134429.s009.doc]

**Table S2.** List of the thirty five radiocarbon measurements on hippo bones from Akrotiri-*Aetokremnos*

| **sample #** | **strata** | **FN** | **square** | **anatomical part** | **thermal treatment** | **dated fraction** | **14C age** | **error** | **target #** |
| --- | --- | --- | --- | --- | --- | --- | --- | --- | --- |
| AA8B1 | surface | 72 | west scree section | bone fragment | charred | non soluble organics | 9235 | 53 | AA 87168 |
| AA8B2 | surface | 72 | west scree section | bone fragment | calcined | bone apatite | 10485 | 57 | AA 87182 |
| AA13 | 2/4 | 125 | N98E88/87, near Feat 1 | distal humerus fragment | charred | soluble organics | 9923 | 55 | AA 87178 |
| AA13 | 2/4 | 125 | N98E88/87, near Feat 1 | distal humerus fragment | charred | non soluble organics | 8126 | 50 | AA 87169 |
| AA14B1 | 2 | 208 | N94E91 | proximal tibia | calcined | bone apatite | 10578 | 58 | AA 87183 |
| AA14B2 | 2 | 208 | N94E91 | proximal tibia | calcined | bone apatite | 10395 | 45 | SacA 28895 |
| AA14B3 | 2 | 208 | N94E91 | proximal tibia | charred | non soluble organics | 6347 | 45 | AA 87170 |
| AA16B | 2 | 208 | N94E91 | bone fragment | calcined | bone apatite | 10612 | 58 | AA 87184 |
| AA25D | 4b | 271 | N94E89 | tooth dentine | unburnt | dentine apatite | 10075 | 45 | SacA 28882 |
| AA25E1b | 4b | 271 | N94E89 | tooth enamel | unburnt | enamel apatite | 10565 | 45 | SacA 28887 |
| AA25E2 | 4b | 271 | N94E89 | tooth enamel | unburnt | enamel apatite | 10540 | 50 | SacA 28881 |
| AA27E1b | 4b | 271 | N94E89 | tooth enamel | unburnt | enamel apatite | 9540 | 40 | SacA 28889 |
| AA27E2 | 4b | 271 | N94E89 | tooth enamel | unburnt | enamel apatite | 9450 | 40 | SacA 28883 |
| AA29D | 4b | 374 | N93E90 | tooth dentine | charred | non soluble organics | 10173 | 56 | AA 87171 |
| AA29E1b | 4b | 374 | N93E90 | tooth enamel | heated | enamel apatite | 10290 | 45 | SacA 28888 |
| AA30B | 4b | 374 | N93E90 | mandibular bone | charred | soluble organics | 9520 | 53 | AA87179 |
| AA30B | 4b | 374 | N93E90 | mandibular bone | charred | non soluble organics | 8354 | 51 | AA 87172 |
| AA30D | 4b | 374 | N93E90 | tooth dentine | unburnt | dentine apatite | 9030 | 40 | SacA 28891 |
| AA30E1b | 4b | 374 | N93E90 | tooth enamel | unburnt | enamel apatite | 10020 | 45 | SacA 28890 |
| AA33B1 | 4b | 375 | N94E90 | bone fragment | calcined | bone apatite | 10430 | 57 | AA 87185 |
| AA33B2 | 4b | 375 | N94E90 | bone fragment | charred | non soluble organics | 9716 | 54 | AA 87173 |
| AA41B | 4a+b | 684 | N97E88 eastern half | bone fragment | charred | non soluble organics | 10084 | 55 | AA 87174 |
| AA42B1 | 4a+b | 684 | N97E88 eastern half | bone fragment | calcined | bone apatite | 10792 | 58 | AA 88554 |
| AA42B2 | 4a+b | 684 | N97E88 eastern half | bone fragment | calcined | bone apatite | 10835 | 45 | SacA 28894 |
| AA45D | 4a+b | 684 | N97E88 eastern half | tooth dentine | charred | dentine apatite | 9895 | 40 | SacA 28885 |
| AA45D | 4a+b | 684 | N97E88 eastern half | tooth dentine | charred | non soluble organics | 10218 | 58 | AA 87175 |
| AA45E1b | 4a+b | 684 | N97E88 eastern half | tooth enamel | heated | enamel apatite | 10365 | 45 | SacA 28886 |
| AA47B | 4a+b | 684 | N97E88 eastern half | fragment of tibia | charred | soluble organics | 10317 | 61 | AA 87180 |
| AA47B | 4a+b | 684 | N97E88 eastern half | fragment of tibia | charred | non soluble organics | 9663 | 65 | AA 87176 |
| AA48B1 | 4a+b | 684 | N97E88 eastern half | fragment of tibia | calcined | bone apatite | 10393 | 57 | AA 88553 |
| AA48B2 | 4a+b | 684 | N97E88 eastern half | fragment of tibia | calcined | bone apatite | 10470 | 40 | SacA 28892 |
| AA50B1 | 4a+b | 684 | N97E88 eastern half | fragment of humerus | calcined | bone apatite | 10457 | 57 | AA 88552 |
| AA50B2 | 4a+b | 684 | N97E88 eastern half | fragment of humerus | calcined | bone apatite | 10445 | 45 | SacA 28893 |
| AA56B | 4c | 767 | N95E88 | fragment of ulna | charred | soluble organics | 10264 | 58 | AA 87181 |
| AA56B | 4c | 767 | N95E88 | fragment of ulna | charred | non soluble organics | 9969 | 61 | AA 87177 |
