# Supplementary material for: Direct Dating and Physico-Chemical Analyses Cast Doubts on the Coexistence of Humans and Dwarf Hippos in Cyprus
Source: PLoS One. 2015 Aug 18;10(8):e0134429. doi: 10.1371/journal.pone.0134429 (PMC4540316; doi:10.1371/journal.pone.0134429)

**Figure S3.** Vis spectra showing the presence of hypomanganate ions (Mn5+) in the apatite structure. A reference Mn-bearing fluorapatite spectrum is shown for comparison. The large absorption band in the region of 600 to 700 nm corresponds to the electronic transition (^3^A_2_🡪 ^3^T_1_ (^3^F)) of Mn5+ ions due to the absorption of red light.


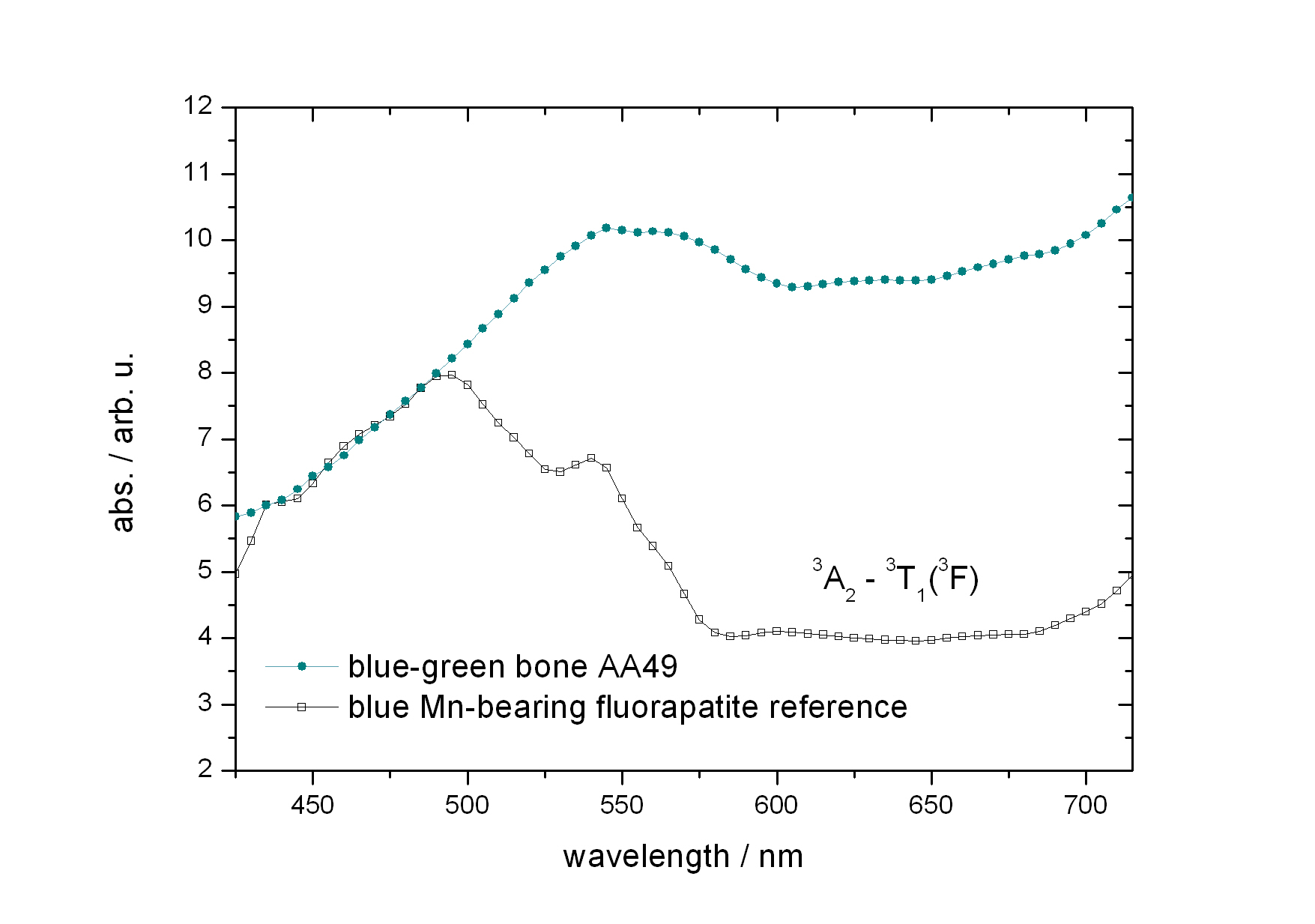

Supplement: S3 Fig — (DOC) [file pone.0134429.s012.doc]
