# Supplementary material for: Direct Dating and Physico-Chemical Analyses Cast Doubts on the Coexistence of Humans and Dwarf Hippos in Cyprus
Source: PLoS One. 2015 Aug 18;10(8):e0134429. doi: 10.1371/journal.pone.0134429 (PMC4540316; doi:10.1371/journal.pone.0134429)

**Figure S4.** a) Optical view of the cross section of sample AA50 and b) localization of elemental composition map. c) Distribution of manganese content over AA50 cross section. Manganese is mainly localized in the first 500 µm of the surface of the cortical bone.


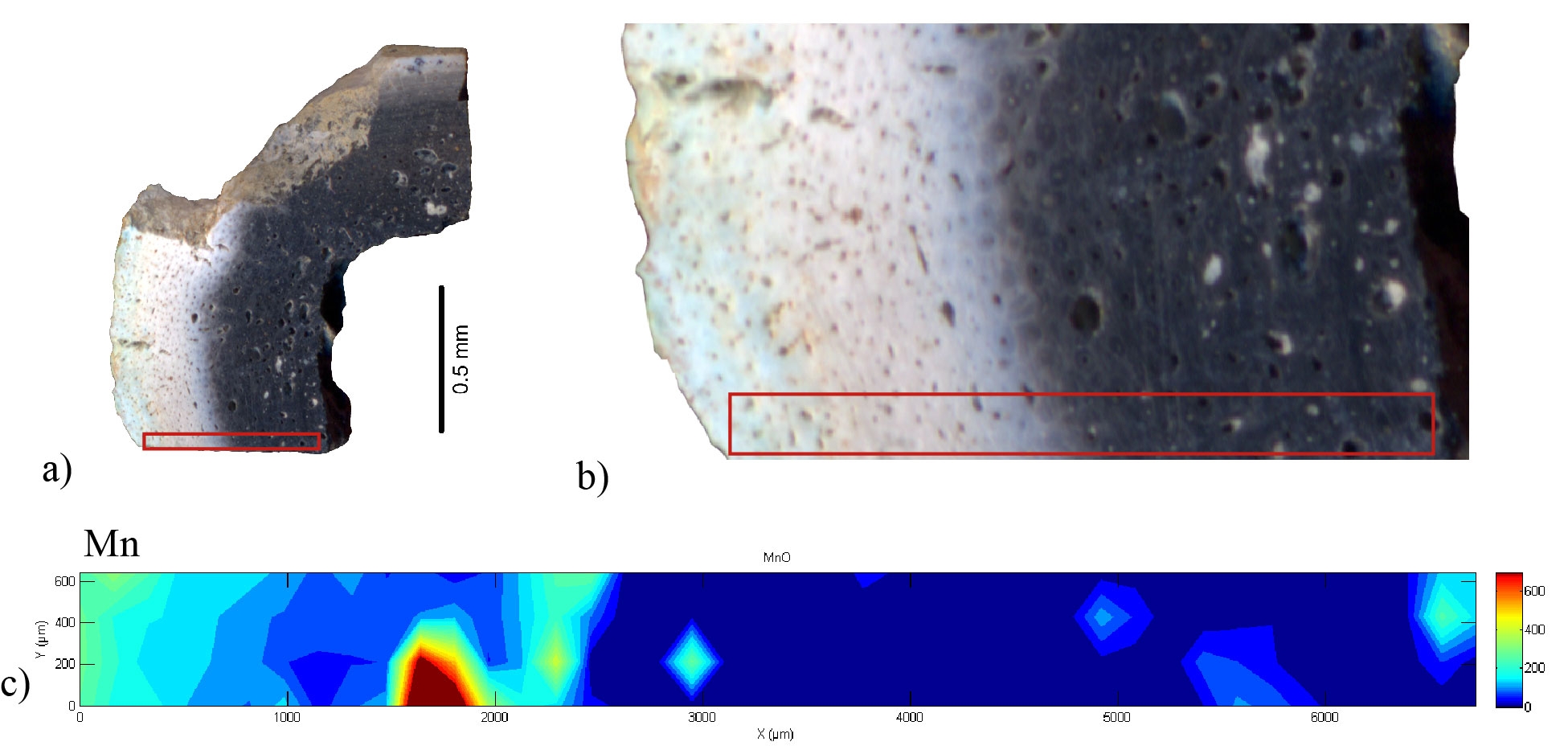

Supplement: S4 Fig — (DOC) [file pone.0134429.s013.doc]
