# Supplementary material for: Direct Dating and Physico-Chemical Analyses Cast Doubts on the Coexistence of Humans and Dwarf Hippos in Cyprus
Source: PLoS One. 2015 Aug 18;10(8):e0134429. doi: 10.1371/journal.pone.0134429 (PMC4540316; doi:10.1371/journal.pone.0134429)

**Figure S5.** Calibrated dates for the thirty five bone samples from Akrotiri-*Aetokremnos*, ranked by decreasing age. Radiocarbon dates were calibrated using OxCal 4.2 (15) and the INTCAL13 calibration curve (16).


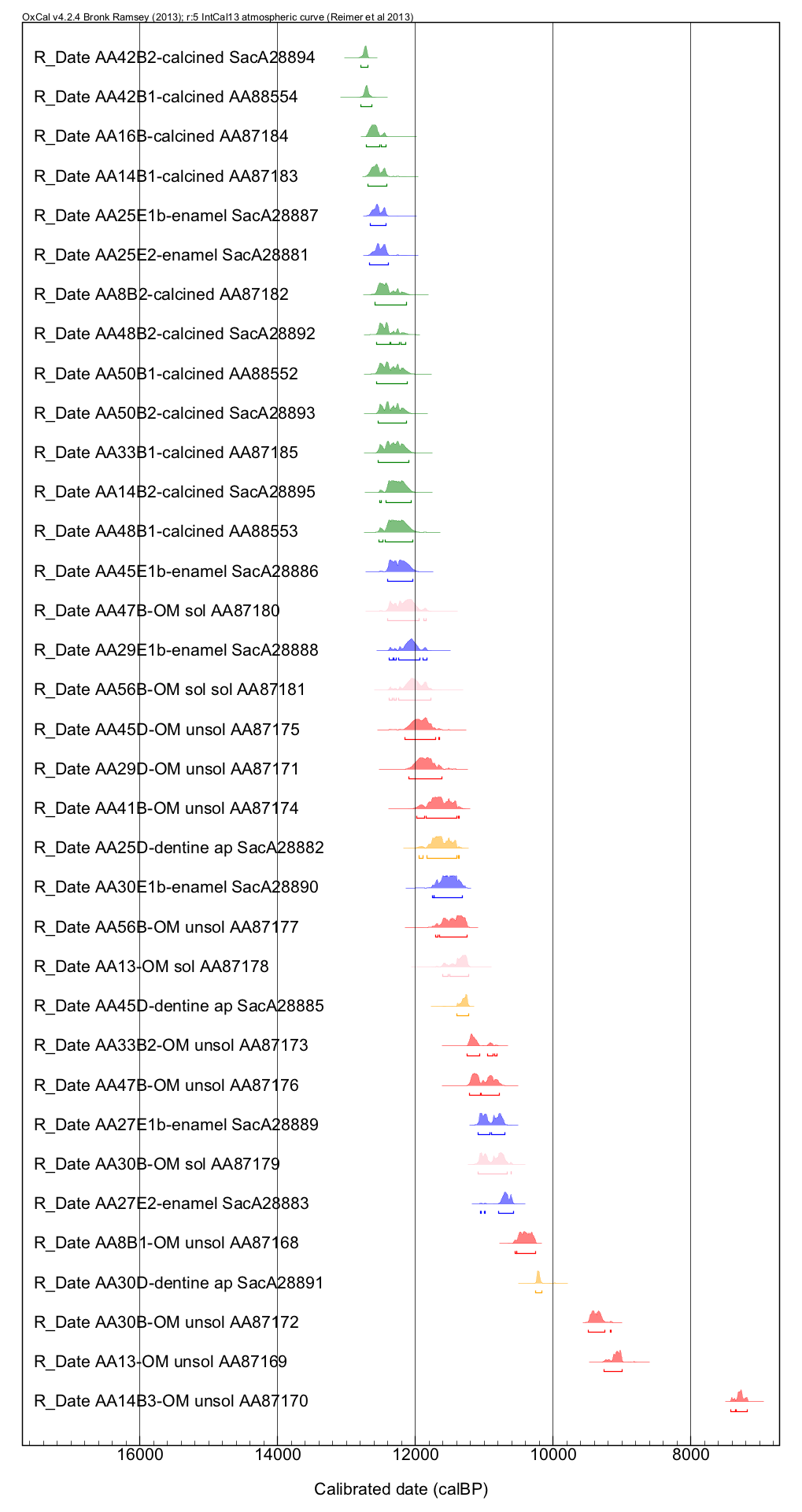

Supplement: S5 Fig — (DOC) [file pone.0134429.s014.doc]
