# Supplementary material for: Direct Dating and Physico-Chemical Analyses Cast Doubts on the Coexistence of Humans and Dwarf Hippos in Cyprus
Source: PLoS One. 2015 Aug 18;10(8):e0134429. doi: 10.1371/journal.pone.0134429 (PMC4540316; doi:10.1371/journal.pone.0134429)

**Figure S6.** Example of intra-individual variability in radiocarbon age measured for different mineral and organic fractions of a partly burnt hippopotamus mandible (AA30). Enamel provided the best approximation of the real age of the sample, followed by soluble organic matter from the degraded collagen in the charred bone, dentine apatite, and unsoluble fraction of the degraded collagen in the charred bone. Radiocarbon dates were calibrated using OxCal 4.2 (15) and the INTCAL13 calibration curve (16).


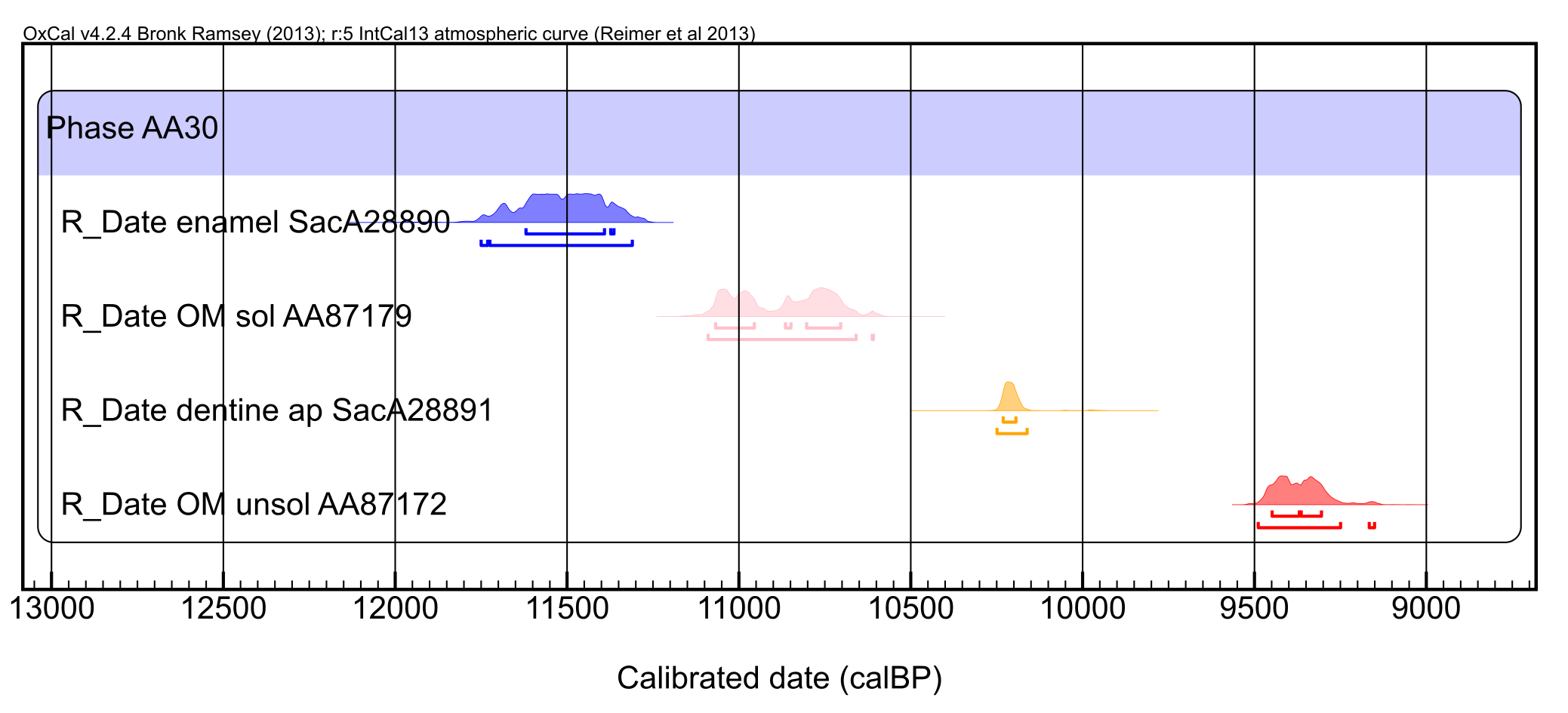

Supplement: S6 Fig — (DOC) [file pone.0134429.s015.doc]
