# Supplementary material for: Direct Dating and Physico-Chemical Analyses Cast Doubts on the Coexistence of Humans and Dwarf Hippos in Cyprus
Source: PLoS One. 2015 Aug 18;10(8):e0134429. doi: 10.1371/journal.pone.0134429 (PMC4540316; doi:10.1371/journal.pone.0134429)
Supplement: S7 Fig — (DOC) [file pone.0134429.s016.doc]

**Figure S7.** First Bayesian model (Model 1) for the radiocarbon dates obtained at the Akrotiri-*Aetokremnos* sequence. The model is built based on the known stratigraphic sequence for the site, and the data are divided into two successive phases: stratum 4 (in red) and stratum 2 (in green). It considers that the hippo bones from stratum 2 were actually deposited together with this stratum. Implying that the hippo survived until the date of the human occupation of the shelter dated to ca. 12,500 cal BP. Prior to modeling, we manually rejected the bone dates on enamel and dentine apatite as well as the soluble and insoluble organic fractions from burnt bones which were obviously diagenetically altered. The dataset (Table 1) is comprised of six calcined bone samples and the previously published six AMS dates on charcoal (1, 3). We did not consider sample AA8 in this model because it was a surface find and, therefore, out of context. This model was generated using OxCal 4.2 (15) and the INTCAL13 calibration curve (16). Lighter shaded distributions are calibrated radiocarbon likelihoods, whereas darker outline distributions are posterior probabilities. Two charcoal determinations (all in stratum 2) produced a very low agreement index of 3 and 6, respectively. They were removed from the subsequent analysis and the model was rerun. Despite one bone determination with a low agreement of 36 (AA87184), the second iteration resulted in a model with an acceptable agreement of 76. Boundaries for the initial and the final versions of Model 1 are shown in Table 2.

| 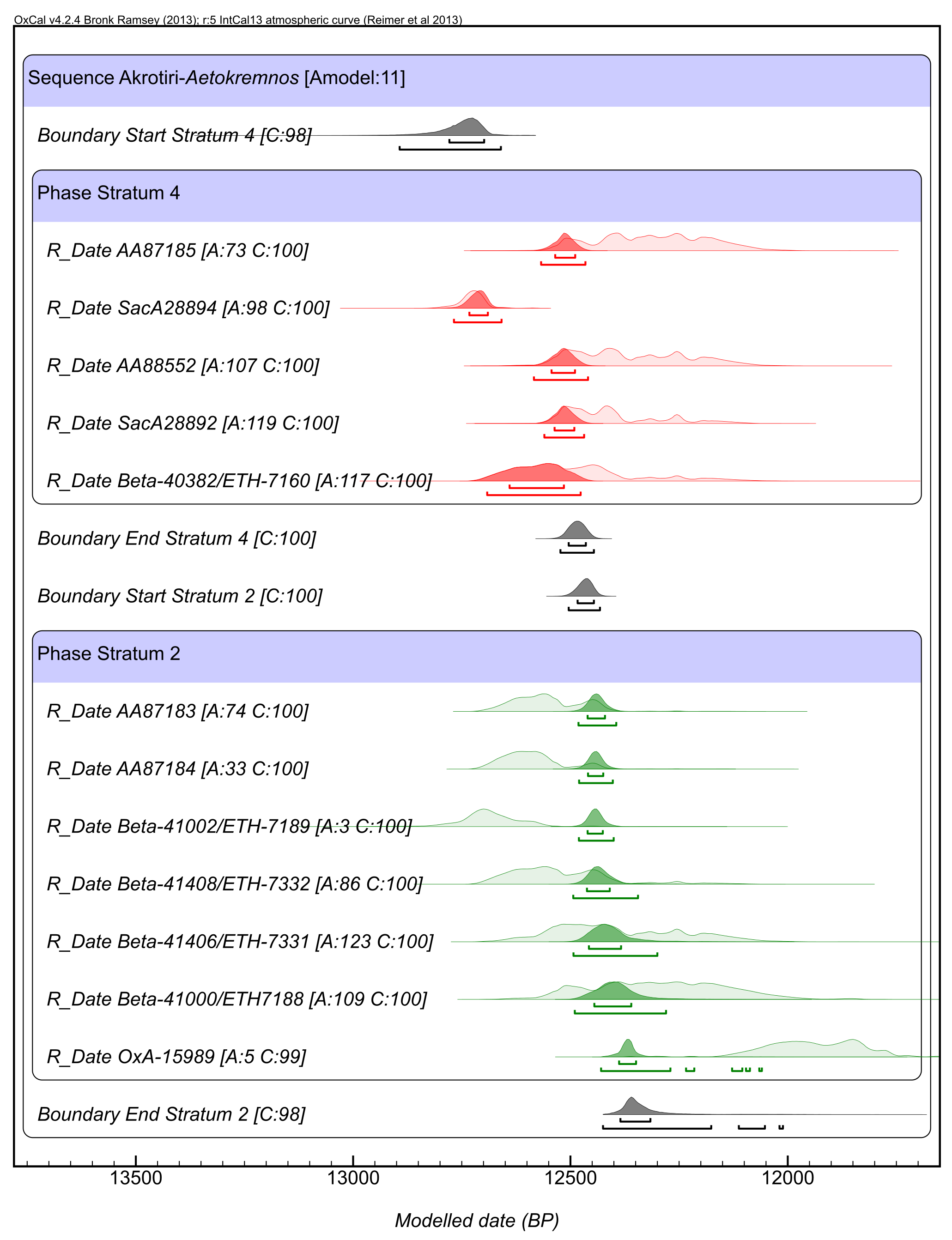 | 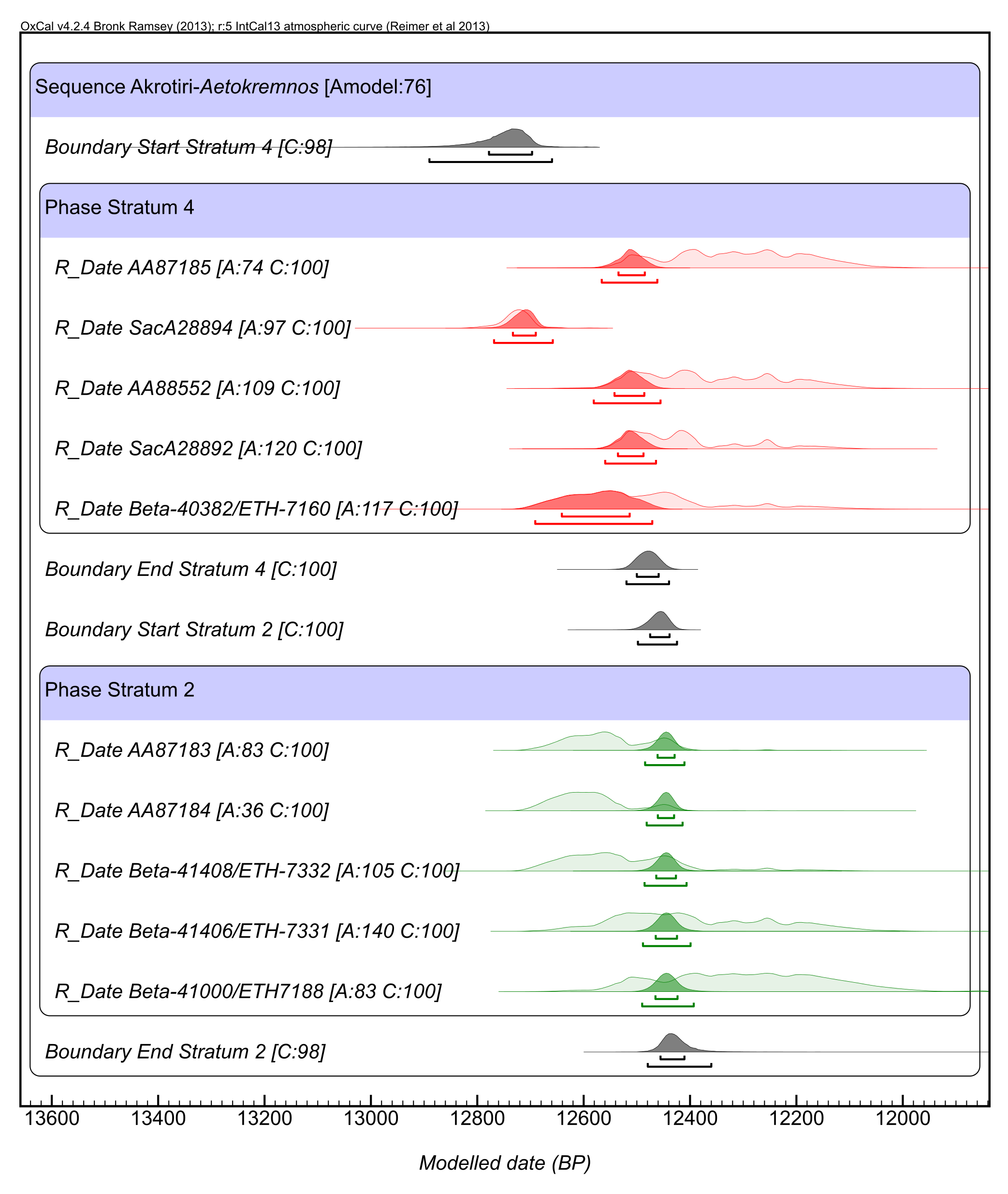 |
| --- | --- |
