# Supplementary material for: Direct Dating and Physico-Chemical Analyses Cast Doubts on the Coexistence of Humans and Dwarf Hippos in Cyprus
Source: PLoS One. 2015 Aug 18;10(8):e0134429. doi: 10.1371/journal.pone.0134429 (PMC4540316; doi:10.1371/journal.pone.0134429)
Supplement: S8 Fig — (DOC) [file pone.0134429.s017.doc]

**Figure S8.** Second Bayesian model (Model 2) for the radiocarbon dates obtained at the Akrotiri-*Aetokremnos* sequence. The data are divided into two successive phases: stratum 4 (in red) and stratum 2 (in green). The model considers that all the bones found in upper stratum 2 actually came from stratum 4, implying that extinction took place before the deposition of stratum 2. Therefore, all the hippo bone determinations were grouped in the same phase (stratum 4). Prior to modeling, we manually rejected the bone dates on enamel and dentine apatite as well as the soluble and insoluble organic fractions from burnt bones which were obviously diagenetically altered. The dataset (Table 1) is comprised of seven calcined bone samples and the previously published six AMS dates on charcoal (1, 3). This model was generated using OxCal 4.2 (15) and the INTCAL13 calibration curve (16). Lighter shaded distributions are calibrated radiocarbon likelihoods, whereas darker outline distributions are posterior probabilities. One charcoal determination (in stratum 2) produced a very low agreement index of 2. It was removed from the subsequent analysis and the model was rerun. The agreement was still low and another charcoal was removed (Beta41408/ETH7332). The third iteration resulted in a model with an acceptable agreement of 86. Boundaries for the initial and the final versions of Model 2 are shown in Table 2.

| 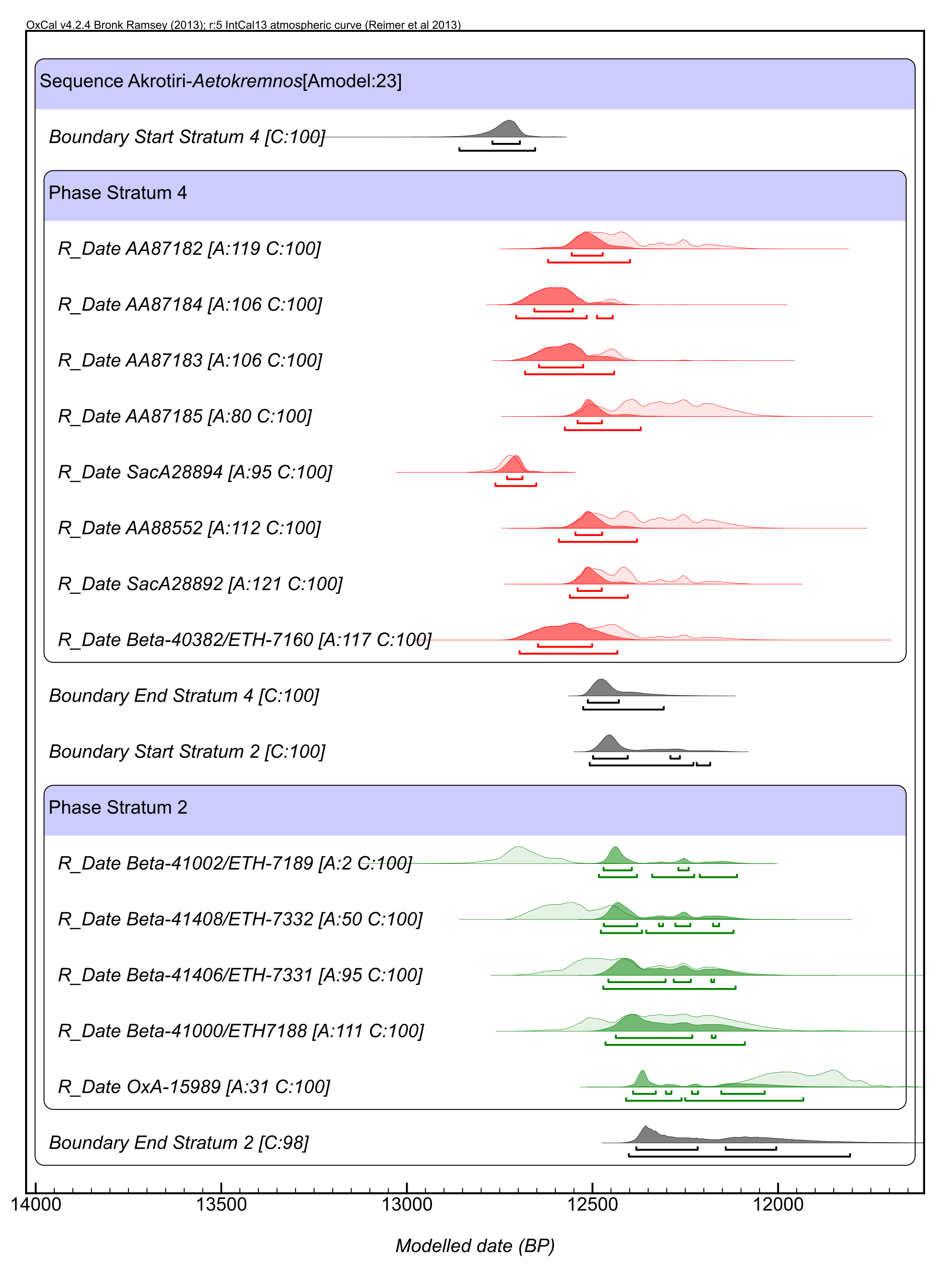 | 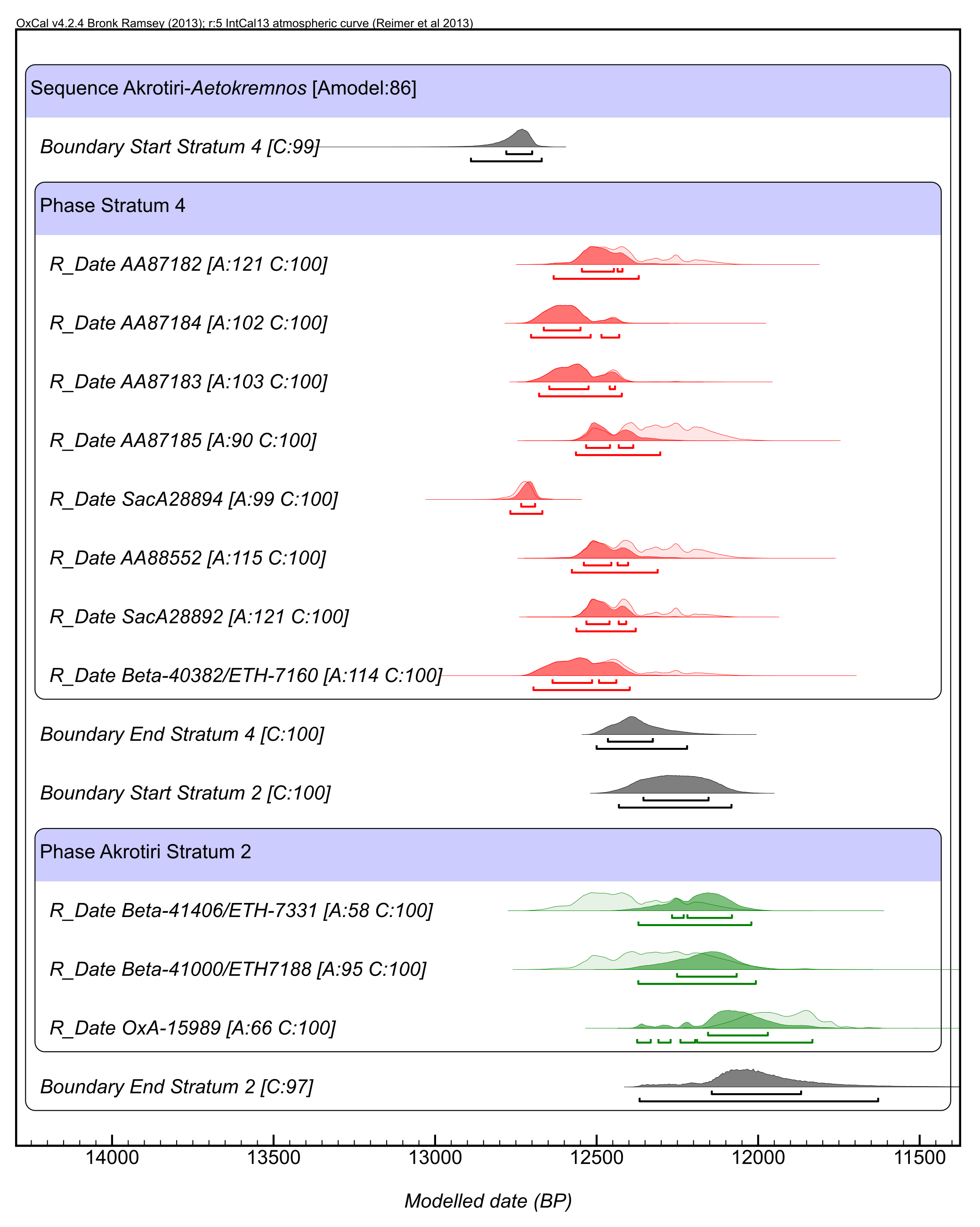 |
| --- | --- |
