# Supplementary material for: Direct Dating and Physico-Chemical Analyses Cast Doubts on the Coexistence of Humans and Dwarf Hippos in Cyprus
Source: PLoS One. 2015 Aug 18;10(8):e0134429. doi: 10.1371/journal.pone.0134429 (PMC4540316; doi:10.1371/journal.pone.0134429)
Supplement: S9 Fig — (DOC) [file pone.0134429.s018.doc]

**Figure S9.** Projection along the calibration curve of the calibrated radiocarbon likelihoods (lighter shaded distributions) determinations and posterior probabilities (darker outline distributions) used for Model 2. The model was generated using OxCal 4.2 (15) and the INTCAL13 calibration curve (16). The Younger Dryas plateau at ca. 10,300-10,500 BP causes an increase in the calibrated densities thus explaining the difficulty in separating stratum 2 (in green) and stratum 4 (in red) statistically.

| 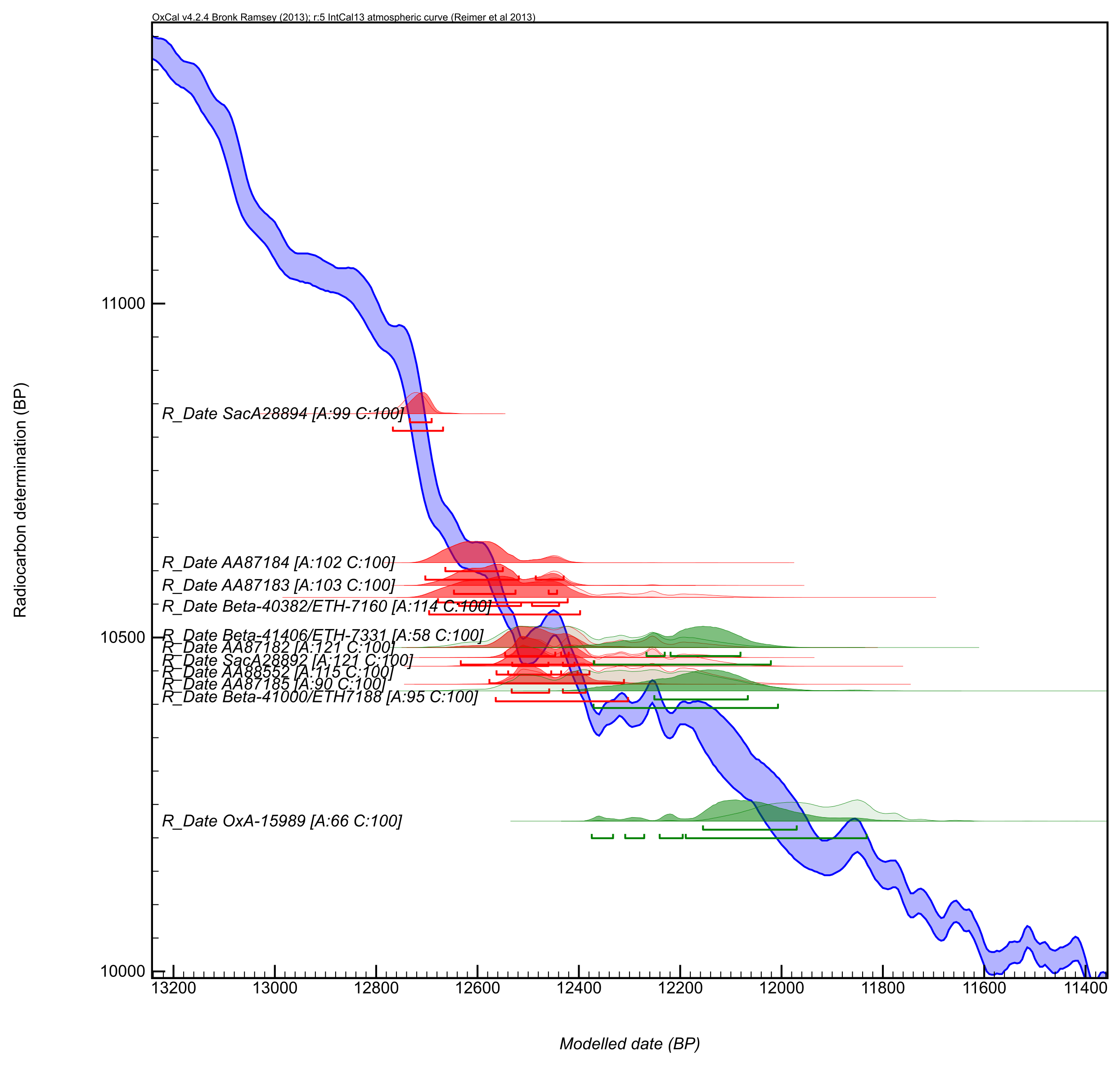 |
| --- |
