# Supplementary material for: Direct Dating and Physico-Chemical Analyses Cast Doubts on the Coexistence of Humans and Dwarf Hippos in Cyprus
Source: PLoS One. 2015 Aug 18;10(8):e0134429. doi: 10.1371/journal.pone.0134429 (PMC4540316; doi:10.1371/journal.pone.0134429)
Supplement: S1 File — (PDF) [file pone.0134429.s001.pdf]

UNLV
UNIVERSITY OF NEVADA LAS VEGAS

Dr. Pavlos Flourentzos
Director, Department of Antiquities
Nicosia, Cyprus

Dear Dr. Flourentzos:

I have had a request for analysis of some bone materials from Akrotiri Aetokremnos. This request is from Dr. Dr. Antoine Zazzo of the CNRS - Muséum national d'Histoire naturelle. As director of the Akrotiri *Aetokremnos* project, I approve of this request. Dr. Zazzo would like to take some small samples of pygmy hippopotamus teeth and other bones for additional radiocarbon dating. As you know, the direct dating of these remains has been an on-going issue, and Dr. Zazzo has access to a very sophisticated radiocarbon laboratory. The further analysis of the bones will go a long way in helping to resolve issues at the site. This will be even more important since we will be doing a small excavation there this summer.

As you know, the materials are curated at the Kourion Museum in Episkopi. Dr. Zazzo would like to have access to these materials in late May of 2009.

Please let me know if any additional information is required.

With my best regards,

Alan H. Simmons

Dr. Alan H. Simmons

Department of Anthropology
Ethnic Studies
4505 Maryland Parkway •Box 455003
Las Vegas, Nevada 89154-5003
(702) 895-3590• Fax (702) 895-4823
http://anthro-ets.unlv.edu/
