# Supplementary material for: Direct Dating and Physico-Chemical Analyses Cast Doubts on the Coexistence of Humans and Dwarf Hippos in Cyprus
Source: PLoS One. 2015 Aug 18;10(8):e0134429. doi: 10.1371/journal.pone.0134429 (PMC4540316; doi:10.1371/journal.pone.0134429)
Supplement: S2 File — (PDF) [file pone.0134429.s002.pdf]

REPUBLIC OF CYPRUS

**MINISTRY OF
COMMUNICATIONS & WORKS**

**DEPARTMENT
OF ANTIQUITIES**
1516 LEFKOSIA

**File no.: 14.01.015.011**
**Tel. no.: 22865807**
**Fax no.: 22303148**

18 March 2009

Dr Allan H. Simmons
University of Nevada
Las Vegas
E-mail: simmonsa@unlv.nevada.edu

Dear Dr Simmons,

I would like to refer to your letter received February 24, 2009 concerning the request from Dr. Antoine Zazzo of the CNRS – *Muséum national d' Histoire naturelle* for analysis of some bone material from Akrotiri Aetokremmos and to inform you that Dr. Dr. Antoine Zazzo has the permission to take some small samples of pygmy hippopotamus teeth and other bones for radiocarbon dating.

Please ask Dr. Zazzo to contact the Archaeological Officer, Mr Yiannis Violaris (25305157) upon his arrival (May 2009).

Yours Sincerely,

(P. Flourentzos)
Director
Department of Antiquities

Copy: Mr Yiannis Violaris
Limassol District Museum

EZ/IN
1401015011
